# An end-to-end convolutional neural network for automated failure localisation and characterisation of 3D interconnects

**DOI:** 10.1038/s41598-023-35048-0

**Published:** 2023-06-09

**Authors:** Priya Paulachan, Jörg Siegert, Ingo Wiesler, Roland Brunner

**Affiliations:** 1 Materials Center Leoben Forschung GmbH, Leoben, Austria; 2 ams-OSRAM AG, Premstaetten, Austria; 3 PVA TePla Analytical Systems GmbH, Westhausen, Germany

**Keywords:** Electrical and electronic engineering, Acoustics, Characterization and analytical techniques, Imaging techniques, Microscopy, Characterization and analytical techniques, Imaging techniques, Microscopy

## Abstract

The advancement in the field of 3D integration circuit technology leads to new challenges for quality assessment of interconnects such as through silicon vias (TSVs) in terms of automated and time-efficient analysis. In this paper, we develop a fully automated high-efficient End-to-End Convolutional Neural Network (CNN) model, utilizing two sequentially linked CNN architectures, suitable to classify and locate thousands of TSVs as well as provide statistical information. In particular, we generate interference patterns of the TSVs by conducting a unique concept of Scanning Acoustic Microscopy (SAM) imaging. Scanning Electron Microscopy (SEM) is used to validate and also disclose the characteristic pattern in the SAM C-scan images. By comparing the model with semi-automated machine learning approaches its outstanding performance is illustrated, indicating a localisation and classification accuracy of 100% and greater than 96%, respectively. The approach is not limited to SAM-image data and presents an important step towards zero defect strategies.

## Introduction

Imaging-based techniques are highly important for modern non-destructive failure analytics^1^ in various fields ranging from aerospace, rail-track inspection, civil engineering, automotive industry, power generation to microelectronics^2^. Machine Learning (ML) algorithms provide novel opportunities for an efficient failure analysis of the generated complex data sets that previously relied mainly on human expertise^3^. Recently, research has been conducted^4–7^ by applying various ML models in 3D integration components which enjoy high interest in microelectronics industry. Essential for the application within an industrial environment are fully automated models, which do not necessarily rely on specific training features. Recently, first effort has been made for the application of ML-based testing as shown in^4,6,7^. Here, mainly semi-automated approaches, have been demonstrated so far. However, such approaches lack in their application for a generalized analysis due to the necessary specific feature definition for the training. Supervised semi-automated ML models like K—Nearest Neighbours (KNN) and a Random Forest classifier are used for example to detect voids in through silicon vias (TSVs) processed in 3D integrated circuit components^4^. As shown, for instance in^4^, such models use specific feature extraction for training, like the High-Frequency Structural Simulator (HFSS) data for the “TSV with” and “TSV without” void. In^6^ a similar semi-automated approach is used, to identify functional failures including open and short circuits for TSVs. Further in this context general regression neural networks are discussed in^7^ to detect defects in solders using SAM.

The Convolutional Neural Network (CNN) is a well-known deep learning ML architecture capable to extract multi-level features from an image^8^. The main advantage of CNN lies in its ability to recognise patterns or relevant features directly from the raw pixels by exploring temporal and spatial correlation in data without any complex pre-processing^9,10^. That is, no prior specific feature definition is necessary for CNN-based approaches. Recently in^5^, a CNN-based model has been utilized to predict the condition of a single micro-bump after the reflow process based on an image data taken before the reflow process by 3D X-ray tomography.

Modern failure inspection of TSVs demands cost- and time-efficient characterisation of hundreds or even up to thousands of TSVs^11,12^ including the concomitant statistical information, the localisation and status of the individual TSV covering the entire geometry with its bottom and sidewall as well as the classification of the TSV failure. There are various defect types related to TSVs including voids resulting from electroplating^13^, delaminations arising due to thermal expansion mismatch^14^, cracks resulting from global stress in the die warpage^15^ and so on^16,17^. To detect such defects, non-automated laboratories techniques like Scanning Electron Microscopy (SEM), X-ray Computed Tomography (XCT), Emission Microscopy (EMMI) or automated techniques^18,19^ such as Electrical Measurements (EM), Automatic Optical Microscopy (AOM)^20^, and Scanning Acoustic Microscopy (SAM) are mainly used^20^. All these techniques have respective advantages as well as disadvantages that restricts their applications. For instance, EM displays a fast and common method, however fails to localise the failure within the TSVs^20,21^. AOM is mainly suitable to detect bottom defects^20,22^ but fails for defects in the sidewall. SEM provides high-resolution image data for the sidewall and the bottom of the TSVs. Nevertheless, the latter is inadequate for high-throughput inspection and ineligible to provide statistical information, due to the very time-consuming data aquisition^23,24^. μ-XCT or X-ray microscopy (XRM) show limitations with respect to the necessary long scanning times to gain sufficient resolution and statistical output^5,15,25,26^. EMMI can only detect defects with an electrical signature and fails in detecting defects without any electrical signature^27^. Scanning acoustic microscopy (SAM) displays a non-destructive technique^28^ capable to characterise time- and cost-efficiently large areas in the field of microelectronics^29^. Nevertheless, the main challenge of this method lies in the limited resolution and contrast as well as in the post-processing of the generated image data set, namely to extract efficiently knowledge about the location of the individual failure but also about the statistical distribution of the failures within the array including the type of the defect class. This requires in general a careful manual inspection of the collected image data. Such a manual inspection highly depends on the experience of the human user, is therefore subjective and in addition prone to error.

In this paper, we conduct a unique scanning acoustic microscopy (SAM) approach and develop an End-to-End Convolutional Neural Network (E2E-CNN) workflow to (1) efficiently characterise up to thousands of TSV on wafer level including concomitant statistical information, (2) localise faulted and non-faulted TSVs, and (3) classify the individual TSVs according to their degree of fault level. We reveal by comparing the gained SAM-data with correlated scanning electron microscopy (SEM) that the SAM technique is suitable to provide information from the bottom as well as from the sidewall of the TSV. The fully automated E2E-CNN network, using two sequentially linked CNN architectures, assures an accuracy for the detection and classification of the TSVs of 100% and greater than 96%, respectively. Furthermore, we discuss the developed E2E-CNN algorithm with semi-automated binary TSV classification using Multi-Layer Perceptron (MLP), Decision Tree, and Random Forest and show its superiority with respect to time efficiency and accuracy. Notably, the presented novel approach is not limited to SAM-based image data, but rather displays a general approach applicable to other imaging methods e.g. μ-XCT, SEM, optical microscopy etc.

## Results

### Scanning acoustic microscopy—experiment and statistical-relevant image data

**Figure 1:**
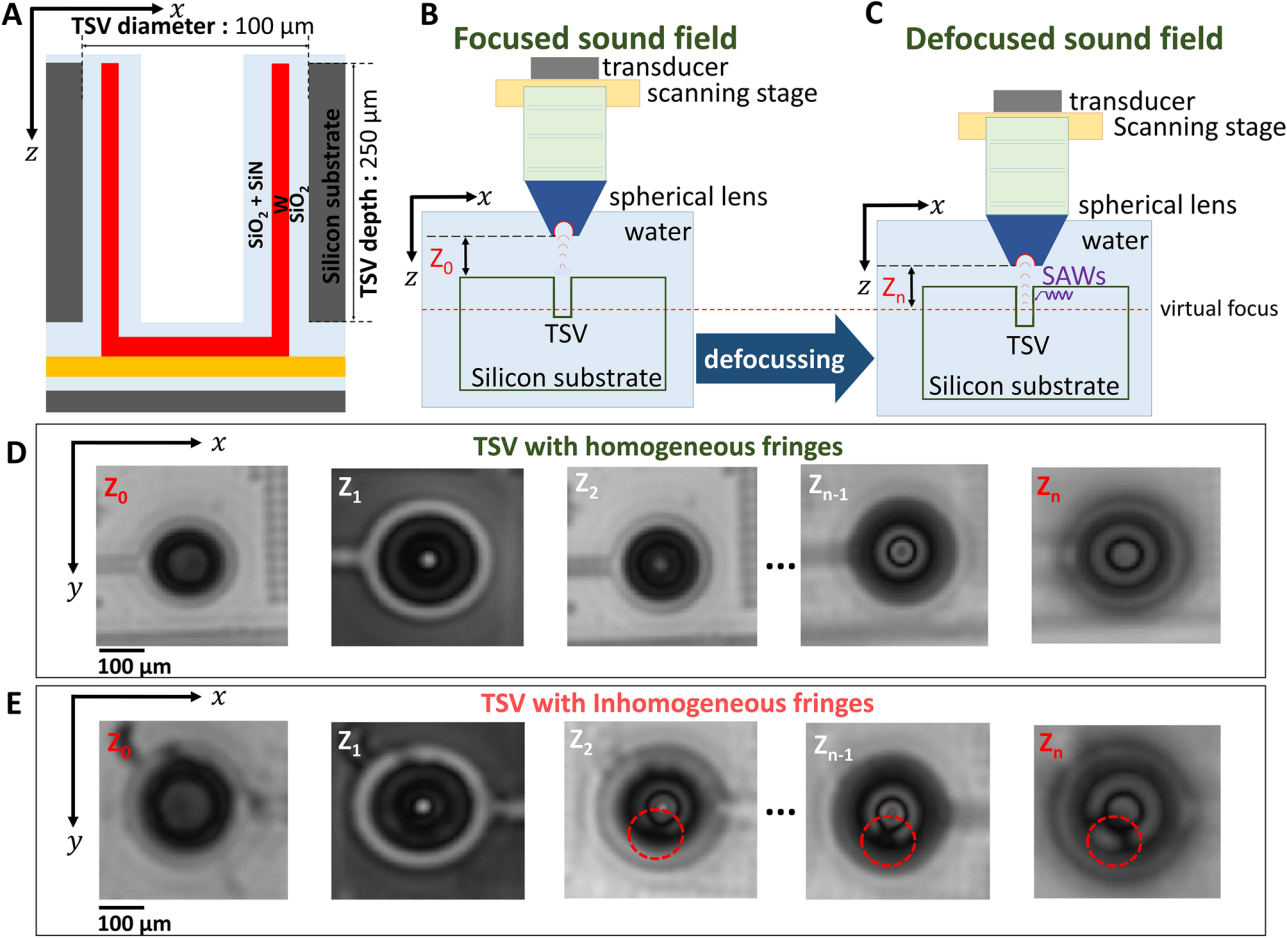
TSV setup and SAM characterisation approach. (**A**) Schematic of an open TSV with a diameter of 100 µm and a depth of 250 µm. (**B**) Schematic of a focused sound field using a Scanning Acoustic Microscope (SAM) at Z_0_. (**C**) Schematic illustrating a defocused sound field at Z_n_. (**D**) SAM C-scan images for different Z-positions starting at Z_0_ for a “good” TSV with no inhomogeneity. (**E**) SAM C-scan images for different Z-positions starting at Z_0_ for a “defected” TSV indicating an inhomogeneity at Z_2_ to Z_n_. Observed inhomogeneity is marked with a red circle.

We apply scanning acoustic microscopy to scan the TSV arrays. Here, we characterise an open TSV technology^46^, see also method section for further sample details and the schematic in Fig. 1A. The major difficulty for defect detection in such TSVs lies in the necessary large penetration depth and resolution in the low μm-regime. We conduct a unique technique utilizing special acoustic lenses with a nominal frequency of 100 MHz and an opening angle of 60°, suitable to fulfill the demands for modern TSV inspection and analysis. The opening angle of the lens in our experiments is chosen in such a way to be larger than the critical Rayleigh angle of Si, which is 17°. The piezoelectric transducer is operating in the pulse-echo mode capable of generating and receiving ultrasound signals. Figure 1B and C shows a schematic for two different lens positions with one position focused (Z_0_) and another defocused (Z_n_) on the surface of the wafer. Figures 1D and E shows the C-scan image of a single TSV with homogeneous and inhomogeneous fringes, respectively obtained at Z = Z_0_ and different defocused positions with Z < Z_0_. The lens at the defocused position provides in the C-scan image patterns or fringes which can be correlated to the TSV quality. An exemplary inhomogeneity observed in the TSV is marked as in Fig. 1E.

The methodology in Fig. 1C represents an extremely time and cost-efficient approach with the ability to collect a statistically relevant amount of TSVs with sufficient resolution for further image post-processing and image quantification. The step size between two transducer positions for the C-scan images depicted in Fig. 1D and E, is 20 µm. A complete Z-series of SAM C-scan image for a single TSV showing an inhomogeneity, is shown in the Supplementary Fig. S1. For the analysis C-scan images at and above Z =  − 120 µm are utilised, see Supplementary Fig. S1.

**Figure 2:**
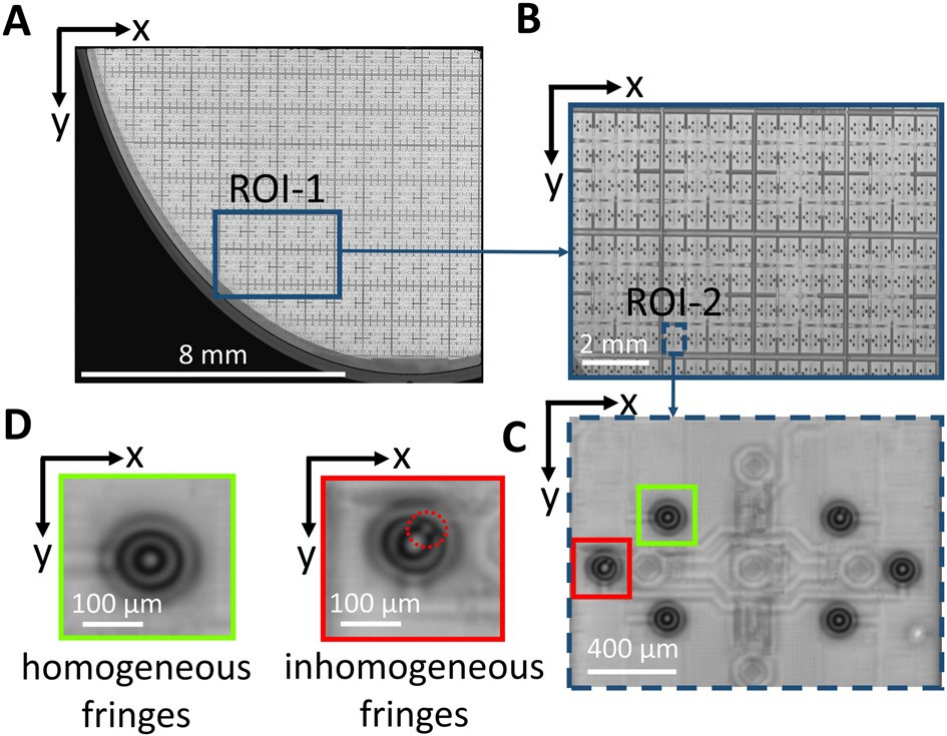
Scanning acoustic microscopy C-scan image data used for testing on wafer-level. (**A**) Overall C-scan image of a quarter-wafer piece by defocusing the acoustic lens with a nominal frequency of 100 MHz and an opening angle of 60° with about 10,000 TSV. (**B**) ROI-1 contains generated patterns within the C-scan of approximately 800 TSVs by defocusing the acoustic lens. (**C**) Zoomed-in C-scan image of ROI-2 with 6 TSVs. (**D**) Characteristic C-scan patterns indicating a TSV with non-disturbed (‘chartreuse’ green rectangular) and disturbed (red rectangular) fringes which may be associated with a good TSV and a TSV implying an inhomogeneity (red dashed circle), respectively.

Figure 2A shows exemplarily the projection of the ultrasound data onto the x–y plane to create the so-called C-scan image of a quarter-wafer piece. The region of interest (ROI) of the C-scan contains approximately 10,000 TSVs. Each TSV can be associated with a characteristic pattern generated by defocusing the lens as shown in Fig. 1D and E. For the better visualisation of the characteristic patterns, the region is divided into a C-scan image patch illustrating about 800 TSVs (ROI-1), as shown in Fig. 2B. It can be further subdivided into an ROI (ROI-2) with six TSVs (Fig. 2C). In Fig. 2D we present exemplarily two patterns generated by defocusing the acoustic lens and exciting Rayleigh waves^30^. The two patterns indicate a TSV without and with an inhomogeneity, marked with a ‘chartreuse’ green and a red rectangle, respectively.

### Workflow of the end-to-end CNN model approach

**Figure 3:**
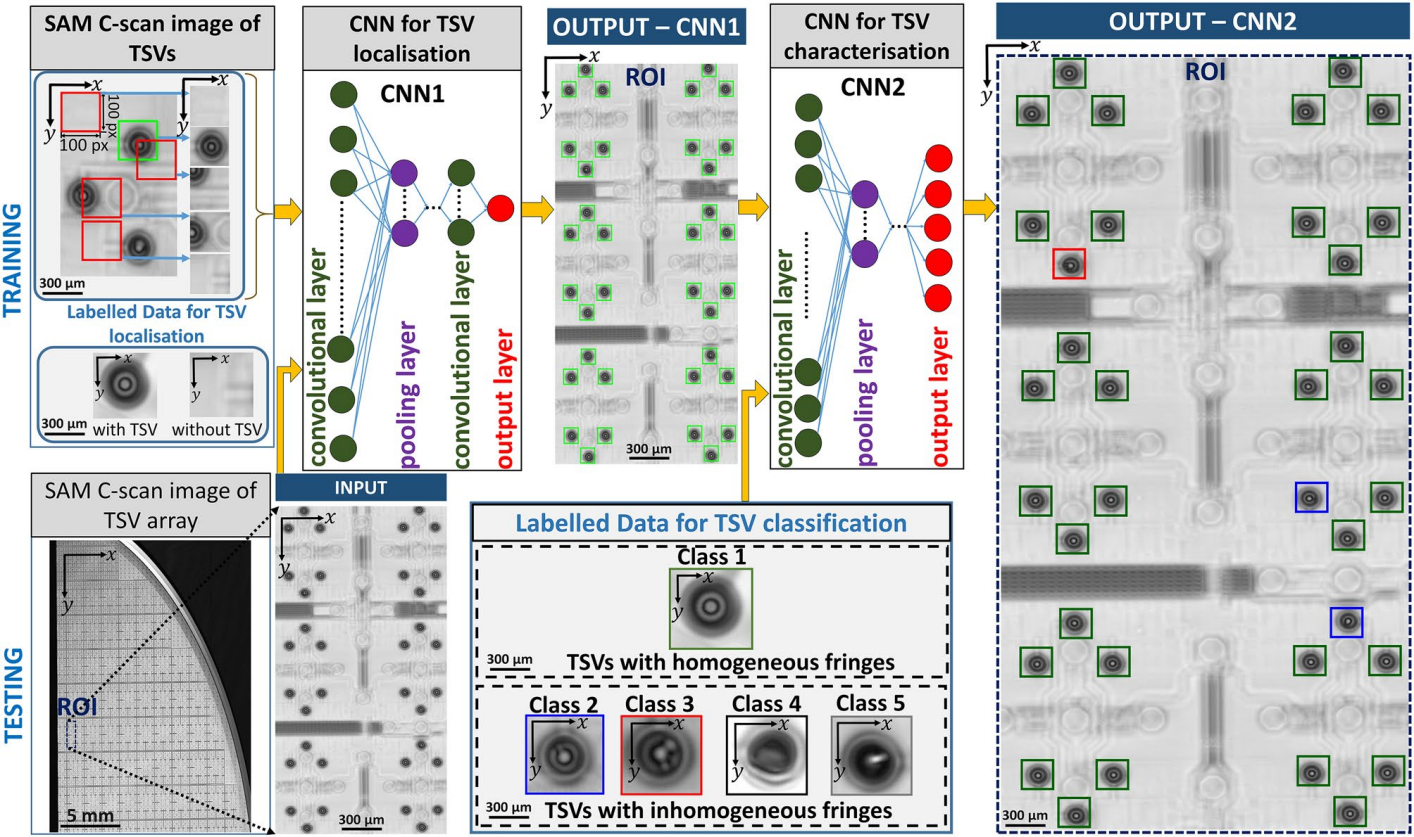
Workflow of automated TSV localisation and classification using the End-to-End Convolutional Neural Network (E2E-CNN). Workflow for training and testing including the architectures of CNN1 and CNN2. SAM-C-scan image data is used as an input in CNN1 for TSV localisation utilising a sliding window TSV detector. Two sets of labelled data indicated by “with” and “without” TSVs (100 × 100 pixel images) are used for the training. CNN2, dedicated for classification, uses the output from CNN1 as an input. CNN2 is trained with five classes defined according to five different patterns found in the C-scan image. The classes are indicated by different colors. The illustrated test image for the quality prediction shows exemplarily 36 TSVs. The intermediate and final predictions of E2E-CNN at a selected rectangular area is marked as OUTPUT—CNN1 and OUTPUT—CNN2, respectively. Localised TSVs in OUTPUT—CNN1 are marked in ‘chartreuse’ color. Predicted classes of TSVs in OUTPUT—CNN2 are indicated by the colors represented by green (class 1), blue (class 2) and red (class 3).

Figure 3 illustrates the automated TSV failure analysis workflow with respect to training and testing, based on the extracted C-Scan SAM data. The workflow consists of two sequentially linked CNN architectures, which we appoint as End-to-End Convolutional Neural Network (E2E-CNN). The first CNN (CNN1) is dedicated to localise the TSVs, whereas the second CNN (CNN2) is capable to classify thousands of TSVs, see further details in the method section.

As an input for CNN1, SAM C-scan image data that is not limited by the image size is used. The output provides image patches with characteristic TSV patterns. CNN2 classifies the TSVs according to their quality and utilizes the output of CNN1 as an input. In order to train CNN1, we use two sets of labelled data incorporating C-scan image patches with and without TSVs, respectively. The output of CNN1 detects all the characteristic patterns which are marked in ‘chartreuse’ color for the exemplary ROI with 36 TSV. Whereas the output of CNN2 is color-coded, based on the quality of the TSVs. We train CNN2 with five different classes indicated by class 1 to 5. The different classes are assigned according to patterns found in the C-scan data. Within the exemplary output of CNN2, shown here, 33 TSVs are assigned to class 1, two to class 2 and one TSV to class 3. Class 4 and 5 is not found in the exemplary ROI.

### Efficient TSV localisation based on a non-sequential sliding window detection for CNN1

**Figure 4:**
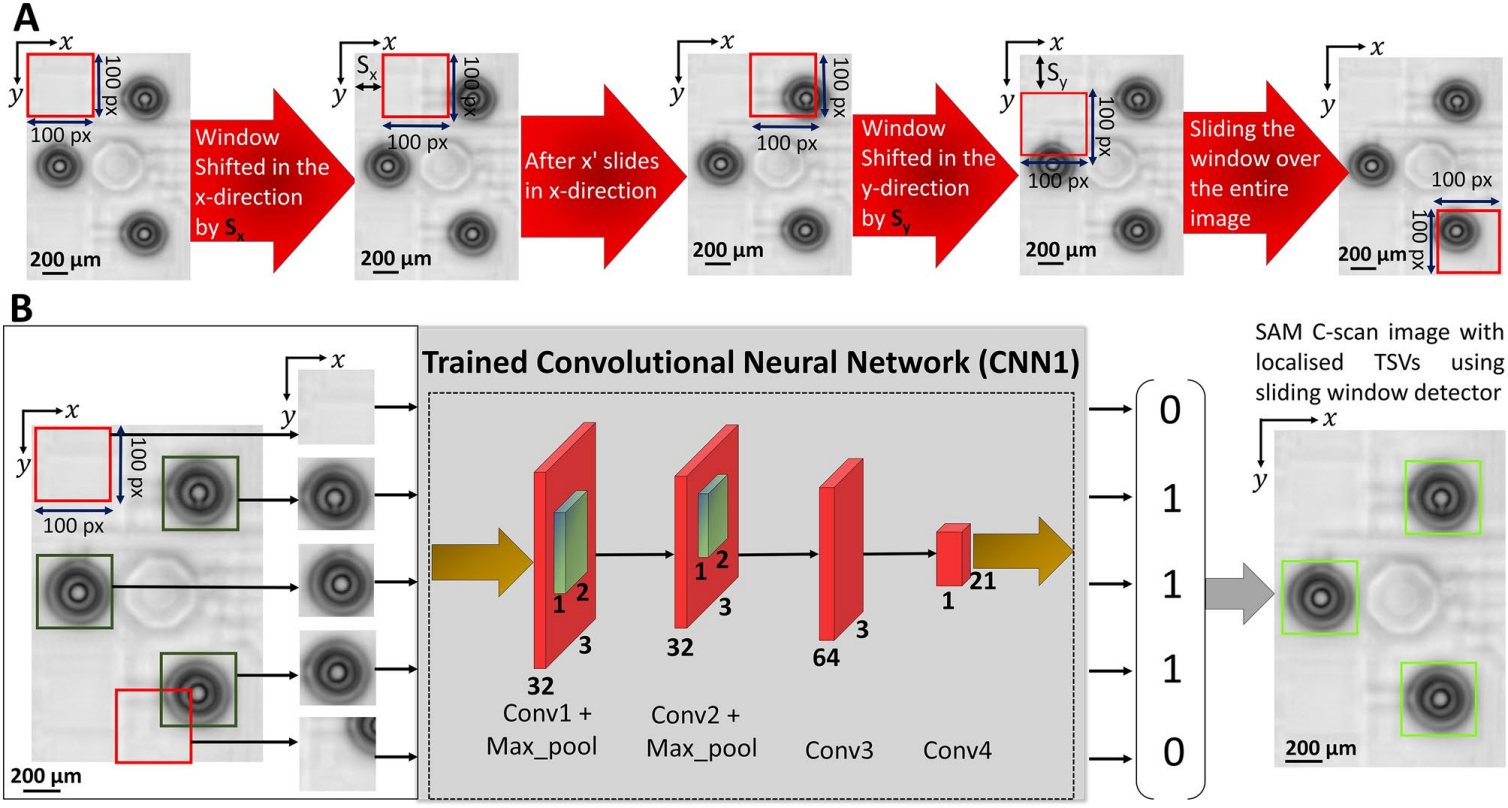
Non-sequential sliding window detector for efficient TSV localisation. (**A**) Schematic of the sliding window procedure in CNN1. Image size of 100 × 100 pixels in x and y directions with strides S_x_ and S_y_ respectively. (**B**) Details with respect to the sliding window detector. The method implements a convolutional layer at the end node, which makes the predictions in a non-sequential manner possible.

There have been several advancements in the field of computer vision for object detections^31^. Many authors proposed object localisation techniques like CNN-based segmentation, sliding window approach and so on^32–34^. Figure 4A shows an illustration for the sliding window detector processing utilised for the TSV localisation. A window with a size of 100 × 100 pixels is chosen to slide over the C-scan image with strides S_x_ and S_y_ in x and y directions, respectively. This specific window size fits well to cover the characteristic patterns of every TSV. For the training of CNN1, each of these windows is individually fed to locate the TSVs in the SAM C-scan images. For the test images two sets are generated. The first set contains the C-scan images of TSVs in the centre of the bounding box. For the second set the image with background and/or any image with TSVs not centred (see Supplementary Fig. S2) are used. Since there are only two categorical features in the dataset for CNN1, by using hot encoding^35,36^, we assign a binary code ‘1’ to the first set and a binary code ‘0’ to the second set while training.

We conduct a non-sequential sliding window detector approach as illustrated in Fig. 4B, see Method section for further details. A major disadvantage of a sequential approach^34^, see also Supplementary materials, Fig. S5, is the computational cost as well as the time consumption for the training and testing result. We show that by using a convolutional layer at the end node^34^ the training time can be reduced from hours to minutes. While the testing procedure takes place, the model predicts multiple bounding boxes^37^ based on whether the extracted features from the window belong to the first set or the second set, i.e. 1 or 0, respectively. Non-Maximum Suppression (NMS) is applied (see Supplementary Fig. S3) to find the predictions with the highest confidence score and gives the best bounding box with a size of 100 × 100 pixels, defining the TSV as an object. The prediction of CNN1 in a C-scan image exemplary with three TSVs is shown in Fig. 4B. Detected TSVs are represented in ‘chartreuse’ colored bounding boxes. The predictions of CNN1 in a larger C-scan image with 864 TSVs is shown in Fig. S4.

### Training of the CNN2 and Input for TSV classification

**Figure 5:**
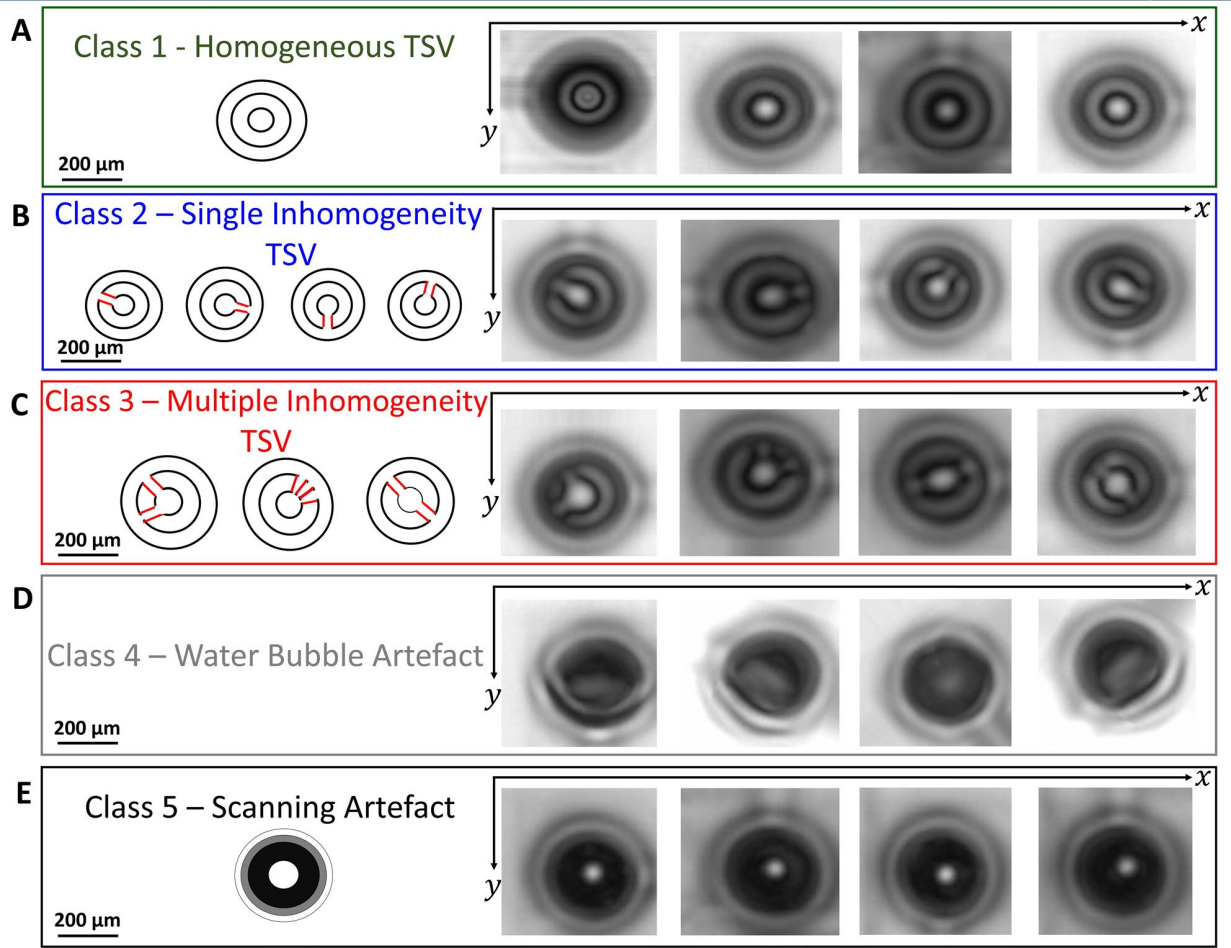
Training data set for TSV classification with five different classes. We define five classes according to the C-scan data. (**A**) Class 1 indicating a circular homogeneous fringe at the position of the TSV. (**B**) Class 2 represents a TSVs with a single inhomogeneity within the circular fringe. (**C**) Class 3 shows exemplarily TSVs with multiple inhomogeneities within the circular fringe. (**D**) Class 4 shows a C-scan image with an artefact resulting from a water bubble. (**E**) Class 5 illustrates a scanning artefact.

The second CNN (CNN2) classifies the located TSVs in the SAM C-scan image. Therefore, the inputs of CNN2 are the predictions from CNN1. According to the C-scan data obtained from the SAM characterisation, we define five classes for the training datasets, see Fig. 5. The first TSV-class exhibits concentric circular or non-disturbed fringes at the TSV location within the C-SAM image, Fig. 5A. The second TSV-class indicate a single inhomogeneity within the circular fringes at various positions along the circumference of the fringes, Fig. 5B. The third TSV-class represents patterns with multiple inhomogeneities along the fringe circumference, Fig. 5C. Class 4 and class 5, shown in Fig. 5D and E represent patterns originating from water bubbles and further scanning artefacts e.g. originating from a gating error or high scanning speed. Further details with respect to the architecture of the CNN2 and corresponding layer parameters are detailed in Supplementary material Fig. S6 and "Method" section.

### Validation of the E2E-CNN

**Figure 6:**
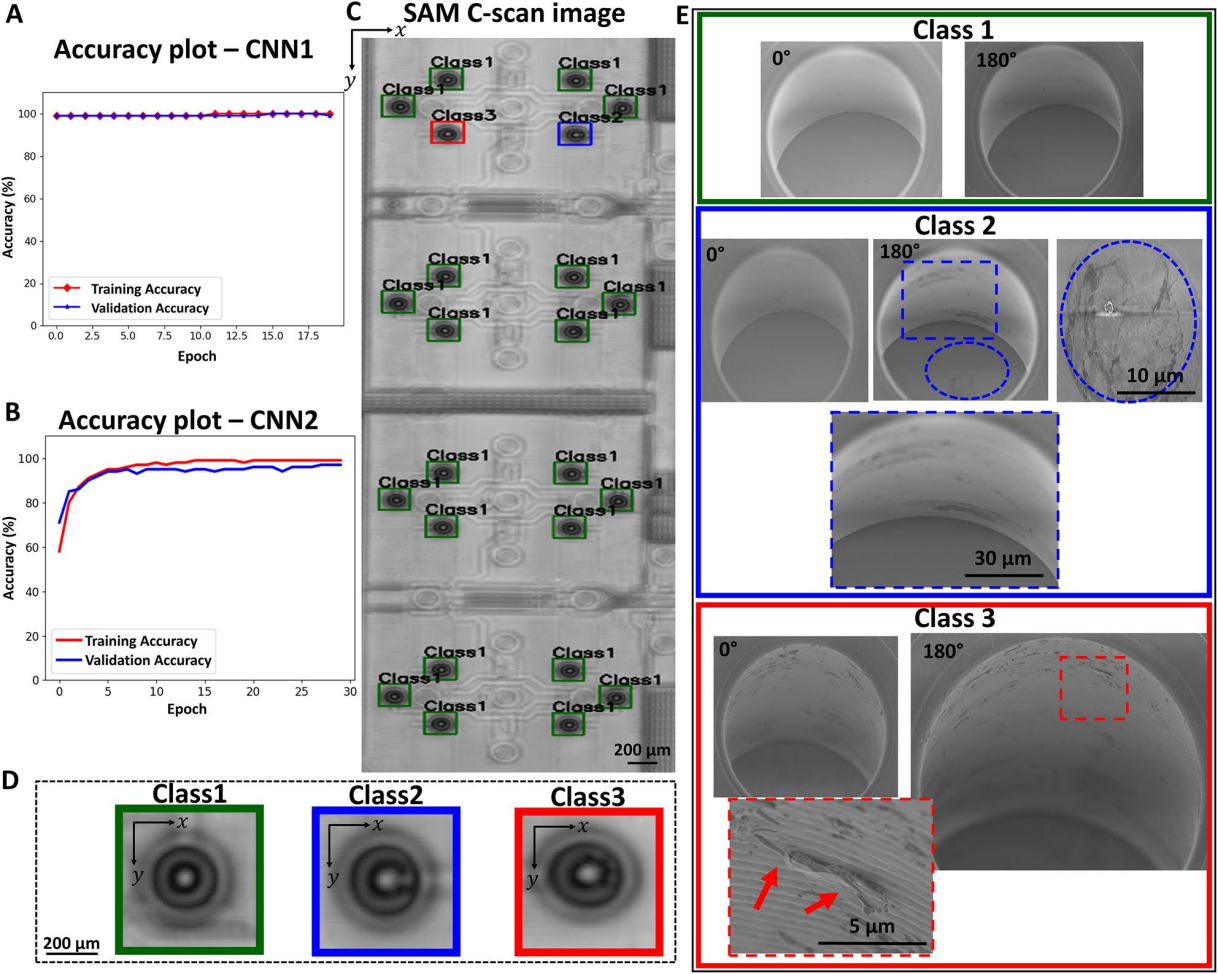
Comparison between SAM C-scan image and SEM data. (**A**) Accuracy plot for the TSV localization (CNN1) including the training (red) and validation (blue)**.** (**B**) Accuracy plot for the TSV classification (CNN2) including the training (red) and validation (blue). (**C**) Localised and classified TSVs in the SAM C-scan image utilising the E2E-CNN prediction. Colored boxes indicate localised TSVs and corresponding assigned classes. (**D**) Zoomed-in images for TSVs indicating class 1 (green), 2 (blue) and 3 (red). (**E**) Representative correlated SEM images with different views and magnifications to gain a comprehensive look from the sidewall of the TSV as well as from the bottom for class 1 (no inhomogeneity), 2 (inhomogeneity at the sidewall and bottom) and 3 (inhomogeneity at the sidewall).

Figure 6A and B illustrate the training and validation accuracy for the two CNN models. For the TSV-localisation (CNN1) we achieve an accuracy of 100% for the validation and training, that is we are able to detect every TSV from the SAM C-scan image, see Fig. 6A. Figure 6B provides an accuracy for CNN2 of greater than 96% dedicated for the TSV-classification alone with respect to training and validation. Further, to show the performance of the E2E-CNN model, we plot the training and validation loss as a function of epochs for CNN1 and CNN2 in Supplementary Fig. S7.

In Fig. 6C a representative SAM C-scan image displays the fully automated localisation and classification of TSVs, exemplarily for class 1, 2 and 3. Images for class 1–3 with higher magnification are shown in Fig. 6D, indicating the different patterns also shown in Fig. 5. For further validation, we compare the SAM C-scan images for class 1, 2 and 3 with correlated SEM characterisation results. As indicated by the SEM data of class 1 in Fig. 6E, no inhomogeneity on the sidewall of the TSV nor at the bottom is detected. This matches with the observation made for the C-scan SAM image where no inhomogeneity in the fringes is exhibited. The SEM data for class 2 shows a large accretion on the bottom of the TSV as well as on the sidewall. Here, a characteristic pattern in the SAM-image indicating a single inhomogeneity within the fringes is shown, see Fig. 6D. For class 3, the SEM data shows a delamination within the sidewall, see Fig. 6E. Here, the C-scan SAM image shows for class 3 a pattern with multiple inhomogeneities in the fringes, see Fig. 6D. According to the correlated SEM data, a clear assignment of the different C-scan patterns can be made.

The detection and quality prediction of 864 TSVs from SAM C-scan images from the wafer is shown in the supplementary Fig. S8.

### Comparison of model performance between automated E2E-CNN and semi-automated ML models

In the following, we compare the developed E2E-CNN model with the semi-automated ML-models. For the semi-automated models, we utilize MLP, Decision Tree and Random Forest, as shown in Table 1. For the semi-automated analysis, to detect the TSVs, it is necessary to apply a geometry based pattern recognition algorithm like the circular Hough transform^38,39^. The data labelling applied for the training and feature extraction steps are the same for MLP, Decision Tree and Random Forest. For the training of the semi-automated ML model, we define two TSV configurations. The first configuration shows TSVs with non-disturbed fringes and the second one TSVs with disturbed fringes in the SAM C-scan images, see Supplementary Fig. S9.

**Table 1:** Comparison of TSV classification performance using E2E-CNN and semi-automated TSV classification models.

ML model name	Semi-automated TSV classification						Fully-automated TSV classification
	Approach 1—CED			Approach 2—FriST			E2E-CNN
	MLP	DT	RF	MLP	DT	RF	
Misclassified TSVs	25	21	31	8	20	24	1
Accuracy (%) to 95% confidence	> 65.0	> 69.7	> 57.6	> 85.2	> 70.8	> 66.3	> 95.9
Pre-processing	Gray scale conversion			Pixel brightness transformations			Augmentation—a total of 537 images are augmented
Manual feature extraction	Canny Edge Detection (CED) Principal Component Analysis (PCA)			Fringe Segmentation Technique (FriST) Principal Component Analysis (PCA)			None
Training data size	3044, 100 × 100 images			3044, 100 × 100 images			5151, 100 × 100 images(CNN2) 14,000, 100 × 100 images(CNNl)
Validation data size	913, 100 × 100 images			913, 100 × 100 images			1714, 100 × 100 images(CNN2) 6000, 100 × 100 images(CNNl)
Testing data size	96, 100 × 100 images			96, 100 × 100 images			96, 100 × 100 images
Time: training	10–15 min			15–20 min			53 min and 12 s
Time: testing	15–20 min			15–20 min			52 s

**Figure 7:**
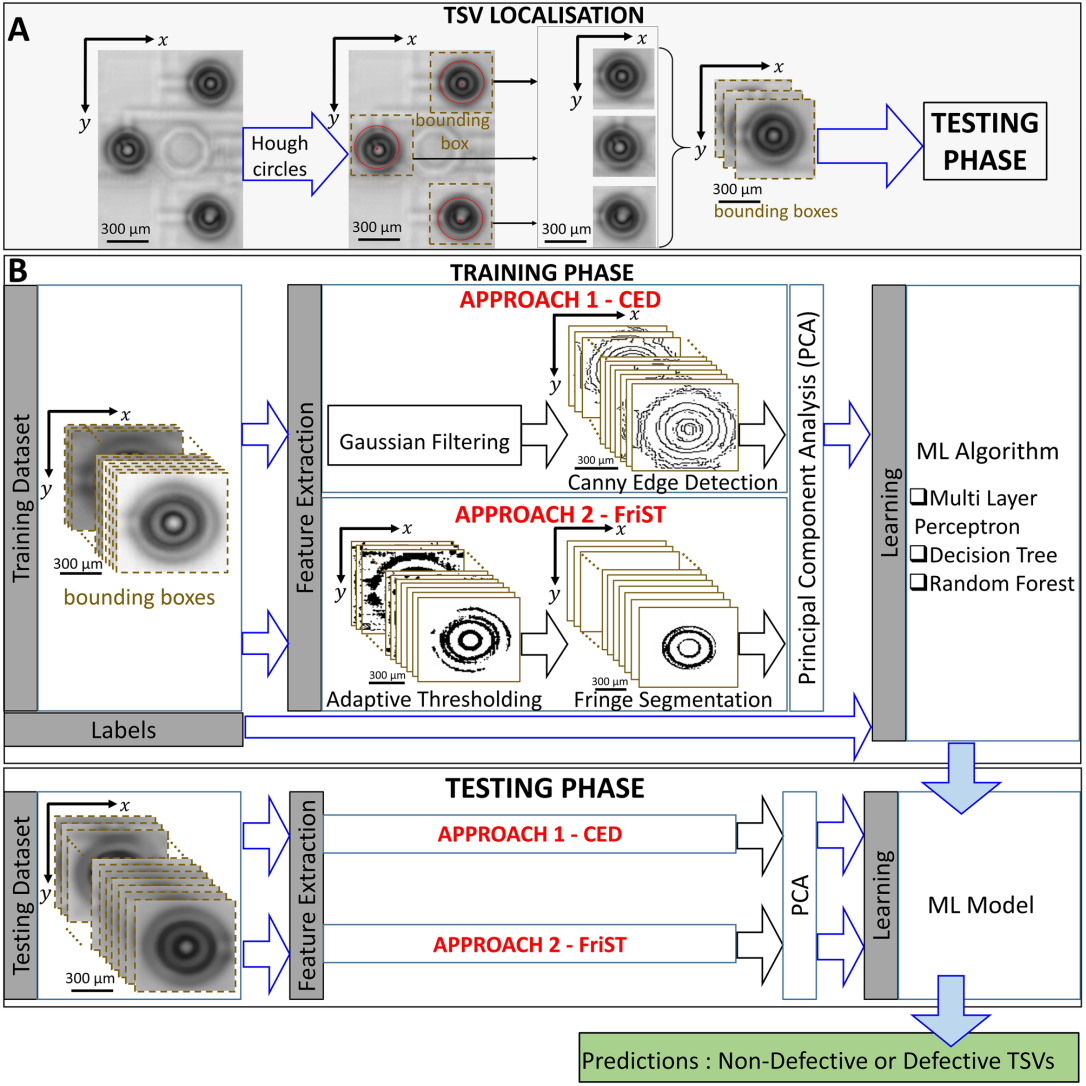
Workflow of semi-automated binary classification of TSV using ML models. (**A**) Detection of TSVs via Hough circles in a SAM C-scan image. (**B**) Binary classification of TSVs using extracted features from CED (approach 1) and FriST (approach 2) techniques. As well as testing phase leading to the classification of TSVs as non-defective or defective TSVs.

Figure 7A illustrates the TSV localisation for the semi-automated ML analysis. Patches with a size of 100 × 100 pixels showing the characteristic patterns are used, followed by the detection of TSVs using circle Hough transform, see Fig. 7A. For the extraction of relevant features we compare two procedures, namely the Canny Edge Detection (CED)^40^ and further developed a unique way of segmentation using the Fringe Segmentation Technique (FriST), see further details in Supplementary Figs. S10, S11 and "Method" section.

For the binary classification of TSVs, the feature extraction using CED or FriST is followed by the dimensionality reduction using Principal Component Analysis (PCA), see Fig. 7B. By applying PCA, we select the most important features from the output of the CED or FriST as an input to train the model, see Methods for further details. The model performance from all investigated models is summarised in Table 1. Note, that due to the limitation of the semi-automated approach with respect to the crucial feature extraction, an analysis of 10.000 TSVs is tremendously time consuming, see also Table 1. Therefore, due to the limited possibilities of the semi-automatic models we down-sized the amount of TSVs to 96 TSVs for the comparison. Further, results for the E2E-CNN with an ROI showing 864 TSVs is presented in the supplementary Fig. S8.

The use of the FriST technique shows for all the three semi-automated models an improvement in accuracy over the CED technique (see Supplementary Fig. S12) due to the specific extraction of desired features for training. However, a general disadvantage for the semi-automated model concerns the requirement for a specific feature extraction to train and test the model. Here, the quality and resolution of the SAM C-scan images is crucial for the subsequent labeling of the pattern associated with the TSV. Therefore, the semi-automated models do not provide an optimal solution when it comes to detecting and classifying large statistics of TSVs, since by increasing the ROI, resolution and contrast will decrease.

Notably, the E2E-CNN workflow in comparison to the semi-automated models does not rely on any manual feature extraction technique. Therefore, the CNN-based approach outperforms the semi-automated ML-based prediction model performance with respect to testing time and accuracy, as shown in Table 1. Indeed, none of the semi-automated models reaches an accuracy of greater than 90% and testing times below 10 min. The key reason for the high efficiency of the E2E-CNN lies in its capability of automatically detecting multiple layers of spatial features from the input image using a set of convolutional operations.

Comparison between the semi-automated ML models (MLP, DT & RF) and the developed fully automated E2E-CNN model for TSV classification from a SAM C-scan image data with 96 TSVs for testing. For the necessary feature extraction of the semi-automated model, we use the CED and the FriST techniques.

### Statistical analysis of SAM C-scan images obtained from the E2E-CNN model

**Figure 8:**
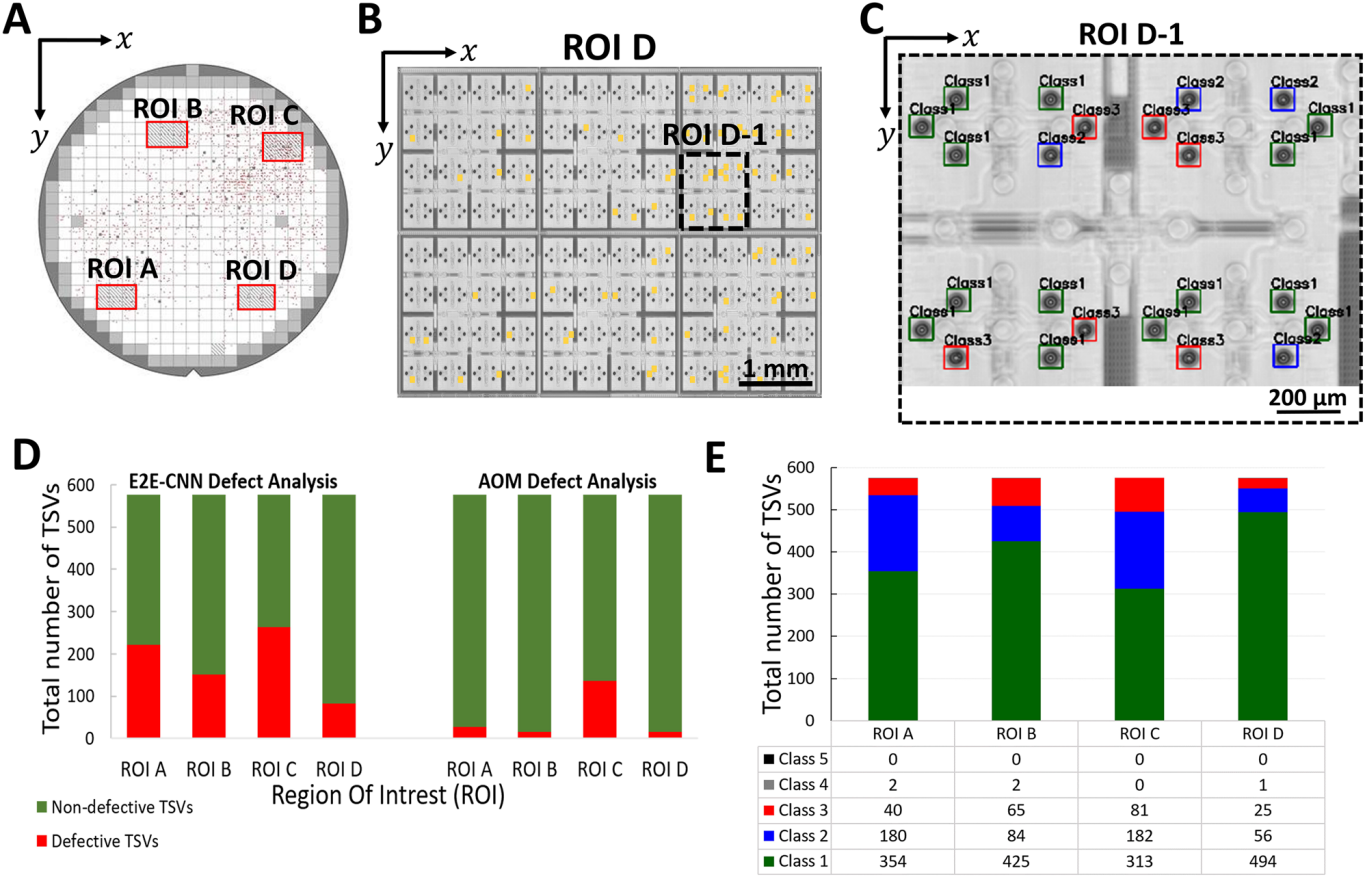
Statistical analysis obtained from the fully automated E2E CNN-based defect analysis and the optical defect analysis. (**A**) Automatic Optical Microscopy (AOM) defect map of the silicon wafer with TSVs. Four ROIs (ROI A to ROI D) are selected for further comparison with the E2E-CNN model. These selected ROIs for defect analysis are marked in red color rectangular boxes. Defects are shown as black dots. (**B**) Defect analysis performed on SAM C-scan image of ROI D using the fully automated E2E-CNN. All inhomogeneous TSVs detected by the E2E-CNN model are marked in yellow color-filled rectangular boxes. The sub region ROI D-1 is highlighted as dashed rectangular boxes. (**C**) Zoomed-in image of ROI D-1. Localised and classified TSVs exhibited by E2E-CNN are marked in green (Class1), blue (Class2) and red (Class3). (**D**) Defect analysis showing the results extracted from the SAM and AOM data for the selected ROIs. The evaluated statistics for TSVs with and without inhomogeneities in the plot are marked in red and green color, respectively. (**E**) Extracted statistics with respect to the different failure classes 1 to 5 for ROI A to D obtained from the E2E-CNN based on the SAM results.

In the following, we utilise the developed E2E-CNN model to highlight the statistical possibilities for the failure analysis. Automatic optical microscopy (AOM) is a conventional, cost-efficient method utilised in wafer inspection. It helps to localise defects within the TSV array by providing two-dimensional defect maps, as shown in Fig. 8A, based on light microscopy. For comparison with the SAM measurements and incorporated E2E-CNN model we select four ROIs, labeled with A, B, C and D at distinct wafer locations. We evaluate the statistics with respect to the occurrence of TSVs with and without inhomogeneities. Each individual ROI selected for the underlying analysis consist of 576 TSVs, see further information in the method section. Figure 8B and C illustrate exemplarily the defect map obtained from the SAM image data as well as the subsequent E2E-CNN analysis for ROI D and a further magnification for ROI D-1, respectively. The latter indicates the TSV location as well as TSV classification according to class 1–5. In this ROI, 568 out of 576 TSVs are classified correctly corresponding to an accuracy of greater than 96%. Further C-scan with prediction results based on the E2E-CNN model for ROI A, ROI B and ROI C are shown in Supplementary Material Fig. S13.

Notably, according to Fig. 8D the extracted statistics illustrates a similar trend for the AOM- and the SAM-based method. ROI C shows for both approaches the highest count with inhomogeneous TSVs and ROI D indicates the lowest one. The depicted results are summarized in Table 2. However, the SAM-based inspection utilizing the E2E-CNN model depicts a higher number of TSVs with inhomogeneities than the optical inspection.

**Table 2:** Statistics showing the different classes 1 to 5 extracted from the E2E-CNN model for ROI A, ROI B, ROI C and ROI D in comparison with the AOM inspection result.

Region of interest	E2E-CNN defect analysis		AOM defect analysis	
	Defective TSVs	Non-defective TSVs	Defective TSVs	Non-defective TSVs
ROI A	222	354	28	548
ROI B	151	425	16	560
ROI C	263	313	136	440
ROI D	82	494	16	560

Further, we provide in Fig. 8E statistical information with respect to different classes predicted by the E2E-CNN model for different ROIs labeled with A to D. Class 2 as indicated by Fig. 6 shows a mixture of sidewall and bottom defects and displays the class with the highest defect count. Usually AOM is suitable to detect bottom defects and is error prone to side wall defects^20,22^. However, the difference between the AOM and SAM-E2E-CNN data as shown in Fig. 8D cannot be fully explained by an inaccurate sidewall detection since class 3, which mainly reflects defects in the sidewall, contains only about 10–20% of the total defects predicted by the E2E-CNN model.

We argue that the higher defect count shown for the acoustic approach is mainly due to the increased detection sensitivity. That is, according to the findings the generated acoustic waves within the TSV lead to a strong interaction with inhomogeneities present at the bottom and sidewalls.

## Conclusion

In conclusion, we have developed a convolutional neural network-based workflow enabling the (1) characterisation of thousands of TSVs on wafer level, (2) localisation of defective TSVs, (3) classification of the individual TSVs according to their degree of defect level and generation of statistical information about the classified TSVs. We use a unique SAM approach to generate cost and time efficiently the image data of the industrial relevant TSV arrays consisting of up to thousands of TSVs. The SAM approach is capable to retrieve information from the bottom and also from the sidewall of the TSV with higher detection sensitivity as shown for AOM. The fully automated E2E-CNN workflow, provides an accuracy for the localisation and classification of 100% and greater than 96%, respectively. Moreover, we explore the possibilities of TSV detection using further machine learning approaches like MLP, Decision Tree and Random Forest models in comparison with the E2E-CNN. Indeed, the analysis shows that those ML approaches cannot compete with the developed E2E-CNN model in terms of time, cost and accuracy. Training as well as testing time for the semi-automated models are time consuming, because specific feature extraction procedures for both training and test images are essential. A major disadvantage is that necessary pattern recognition algorithms strongly depend in general on the quality of the image data. Therefore, sufficient image resolution and contrast to detect the TSV is mandatory. Pre-processing of the gained SAM C-scan image using pixel brightness transformations like histogram equalisation^41^or by applying different thresholding techniques such as adaptive thresholding^42^, binary thresholding^43^ may help to improve the image quality for further processing. However, our high accuracy model eliminates any time-consuming requirement for manual inspection or such pre-processing of the C-scan images. This makes the E2E-CNN model highly applicable in analysing larger C-scan images with characteristic patterns of thousands of TSVs. Further, the presented E2E-CNN workflow for automated failure inspection is not limited to the analysis of SAM image data but rather can be applied also for other imaging methods.

## Methods

### Sample

Through Silicon Vias (TSV) are key components for 3D integration technology that plays an important role in miniaturization and improving the functionality of microelectronic devices^12,44^. TSVs enable an electrical connection through different layers of the 3D stacks. These metallized vias are kept by etching holes into the silicon, and subsequently filling or coating with a conductive material – closed and open TSVs, respectively. Even though closed TSV design has very low contact resistance, they suffer from a high degree of mechanical stress due to the mismatch of coefficient of thermal expansion between silicon and the filling material^45^. Therefore, tungsten-lined open TSV technology replaces the closed TSV one when thermal expansion is of particular concern^46^. In this work, we use an open TSV technology with a 100 µm diameter and 250 µm depth. One potential issue associated with an open design is the high residual stress of tungsten after the deposition process, which can lead to cracks, delaminations, or accretions either on the sidewall or on the TSV bottom^45^ depending on the quality of underlying layers. For this research, ams-OSRAM AG, Premstaetten, Austria, provided wafers with artificially induced defects in TSVs by alterations in the normal fabrication process. These TSVs with unknown defects at unknown locations are detected non-destructively using SAM.

### Experimental setup

The SAM used for this study is a modified setup from PVA TePla Analytical Systems GmbH, Germany, with an ADC card of 8-bit resolution and sampling rate of 5 Giga samples/s, and a tone burst configuration with 10 ns burst-length. For obtaining the characteristic pattern using our approach, we utilize a transducer with 100 MHz central frequency and a lens with an opening angle of 60° which capable of exciting Rayleigh waves^30^. The reflections from the sample are analysed using the software ‘WinSAM 8.0.2293.0’ provided by PVA TePla. The scan rate of SAM using our approach is about 1000 TSVs per 45 min with a resolution of 2 µm/pixel.

### Machine learning (ML)

We classify the characteristic C-scan SAM patterns using semi and fully automated ML models. For the fully automated model an End-to-End Convolutional Neural Network (E2E-CNN) with two CNN architectures, indicated by CNN1 and CNN2 is dedicated to localise and classify the TSVs, respectively.

#### Fully automated ML model

CNN1—The architecture of the CNN1 comprises three convolutional layers with 32, 32, 64 filters and two max pooling layers arranged in a stacked sequence, see also supplementary Fig. S7 with respect to the architecture selection process. We used a 2D kernel to extract features from the image at each level from the CNN. Those Kernels perform feature extraction by taking a dot product between sub regions in the image with itself. The first layer of the CNN1 consists of a convolutional layer with kernel size of 3 × 3, which reduces the dimension of the input to 98 × 98 × 32. The second layer is a max-pooling layer with a kernel size of 2 × 2 and stride 2. The third layer is a convolutional layer with another 3 × 3 kernel. This layer reduces the dimension of features to 47 × 47 × 32. The output of the third layer is forwarded to the fourth layer, which is a max-pooling layer. This layer has the same kernel size as the first pooling layer. The fifth layer is again another convolutional layer with kernel size of 3 × 3 and further reduces the dimension of the features to 21 × 21 × 64. The output layer of CNN1 represents a convolutional layer with sigmoid activation function^47^ that predicts in the range of zero to one, depending on whether the TSV is detected or not. The output convolutional layer consists of one filter with a kernel size of 21 × 21. To train CNN1, we use a dataset with overall 20,000 images. We split the entire image data of CNN1 with 70% for the training and 30% for the validation to evaluate the model performance. That is, the training batch and validation batch consists of 14,000 and 6000 images, respectively.

CNN2—It consists of six convolutional layers with 32, 32, 64, 64, 64, 128 filters and three max pooling layers, see also supplementary Fig. S7 with respect to the architecture selection process. The output layer of CNN2 is a fully connected layer with a softmax activation function^47^, which assigns decimal probabilities to each class. The whole dataset of CNN2 consists of 6865 images with 100 × 100 pixel. It shows five output class labels representing the different characteristic patterns illustrated in the C-scan image, Fig. 5. From a dataset of 6865 images, 5151 images are used for training the model and 1,714 images for validating the model. As described above, the input to CNN2 is a C-scan image patch with 10,000 pixel values, i.e. image patches with the characteristic pattern of each individual TSVs. The input is convolved with two 32 3 × 3 filters. This reduces the dimension of input to 96 × 96 × 32. The third layer is a max-pooling layer with stride 2. The following three layers are convolutional layers that are stacked up and learn 64 features through a 3 × 3 filter. The output of these layers is of size 42 × 42 × 64 and this is forwarded to the next max pooling layer. The eighth layer of CNN2 is another convolutional layer that learns 128 features through a 3 × 3 filter. The output of the following max pooling layer is flattened and provides as input to the dense layer with five output nodes. All the convolutional layers of CNN2 go with a stride = 1 and a rectified linear unit (ReLu) activation.

#### Semi automated ML model

The input for the feature extractors are 3044 SAM C-scan images of TSVs with 10,000 pixels, i.e., a bounding box with characteristic pattern of TSVs. This bounding box is obtained from the centre of TSVs detected using Hough circles. Two sets of features are extracted from this image patches using approach 1 (CED) and approach 2 (FriST). For the training of the MLP, DT and RF models as shown in Table 1, we use 70% of total dataset for training the model and 30% of total dataset for validating the model. For the training and validation of the Decision Tree and Random Forest, we extracted the same features as for the MLP using CED and FriST techniques. The modelled MLP consists of an input layer with 1000 nodes, hidden layers with 250, 125, 50 nodes, and one output node. The solver used for weight optimization is Limited Memory Broyden Fletcher Goldfarb Shanno (LBFGS) and cross-entropy for measuring the penalty associated with the predictions (loss function).

Canny Edge Detection (CED): Before extracting any edge information from TSV fringes, we applied the Gaussian filter in order to remove any scanning artefacts or noises present on the image. Then we detected fringe edges from this by applying a CED algorithm. The edge features thus obtained are further narrowed by applying PCA to this data.

Fringe Segmentation Technique (FriST): Here, the bounding box contains the pre-processed characteristic patterns using adaptive thresholding. Then a circle with unit radius is considered at the centre of the pre-processed image. The radius of this circle is increased iteratively until it reaches the end of the bounding box (see supplementary Fig. S10). From the C-scan images, the information concerning the TSVs mainly lies in the inner two fringes. At each step, the total number of black pixels that lies in the circumference of this circle is plotted. The first and the second peaks in the plot correspond to the fringes of interest and therefore, retain those areas of image segments by setting all the pixels lying beyond these two peaks to 255 (see supplementary Figs. S10 and S11). These steps are iterated sequentially for each TSV to extract the relevant features from the fringes. The relevant ROIs segmented using FriST technique is also fed to PCA.

### Image pre- and post-processing

For the E2E-CNN model no pre or post processing of SAM C-scan images is required whereas in semi-automated TSV localisation and classification, depending the quality of SAM C-scan image, we need to prepare the image using various image processing techniques like contrast enhancement, filtering, thresholding and so. 10.000 TSVs can be typically analysed. One image file with 2 μm/pixel shows a file size of 600 MB. Therefore, we select for further analysis ROIs with about 576 TSVs taking the limited computational power into account. For CNN 2, we augmented TSVs with characteristic patterns belonging to class 4 and class 5. Such C-scan images indicate water bubbles and scanning artefacts and are rather rare. The size of the augmented data set together for class 4 and class 5 is 537. For the augmentation we mainly used flipping, rotation, zoom-in and zoom-out.

## Supplementary Information

Supplementary Information.

## Data Availability

All data that support the findings of this study are available from the corresponding author upon reasonable request.

## Code availability

All code that support the findings of this study are available from the corresponding author upon reasonable request.
