# Supplementary material for: An end-to-end convolutional neural network for automated failure localisation and characterisation of 3D interconnects
Source: Sci Rep. 2023 Jun 9;13:9376. doi: 10.1038/s41598-023-35048-0 (PMC10256798; doi:10.1038/s41598-023-35048-0)
Supplement: Supplementary file 1 — Supplementary Information. [file 41598_2023_35048_MOESM1_ESM.docx]

**Supplementary File:**

**An End-to-End Convolutional Neural Network for Automated Failure Localisation and Characterisation of 3D Interconnects**

Priya Paulachan^1^, Jörg Siegert^2^, Ingo Wiesler^3^, Roland Brunner^1*^

*Corresponding author email: [roland.brunner@mcl.at](mailto:roland.brunner@mcl.at)

^1^ Materials Center Leoben Forschung GmbH, Austria

^2^ ams-OSRAM AG, Premstaetten, Austria

^3^ PVA TePla Analytical Systems GmbH, Germany

**
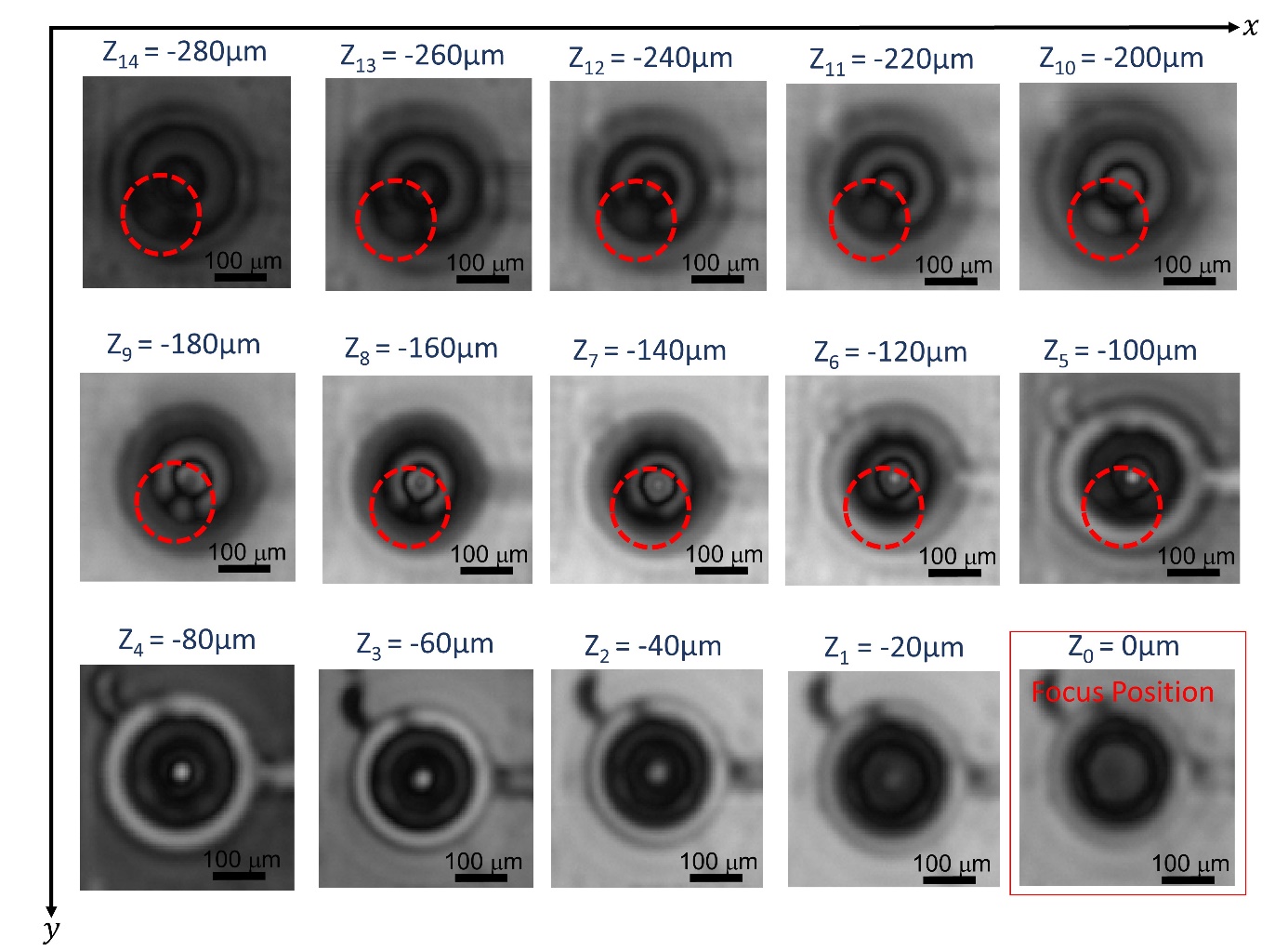
**

**Figure S1.** SAM C-scan images of a single TSV for different z-positions. At about and above z=-120 µm the inhomogeneity within the characteristic pattern is visible. The step size between two z-positions is about 20$\mu m$ (Z_0_ >>Z_14_). The inhomogeneity is highlighted by a red circle.

**
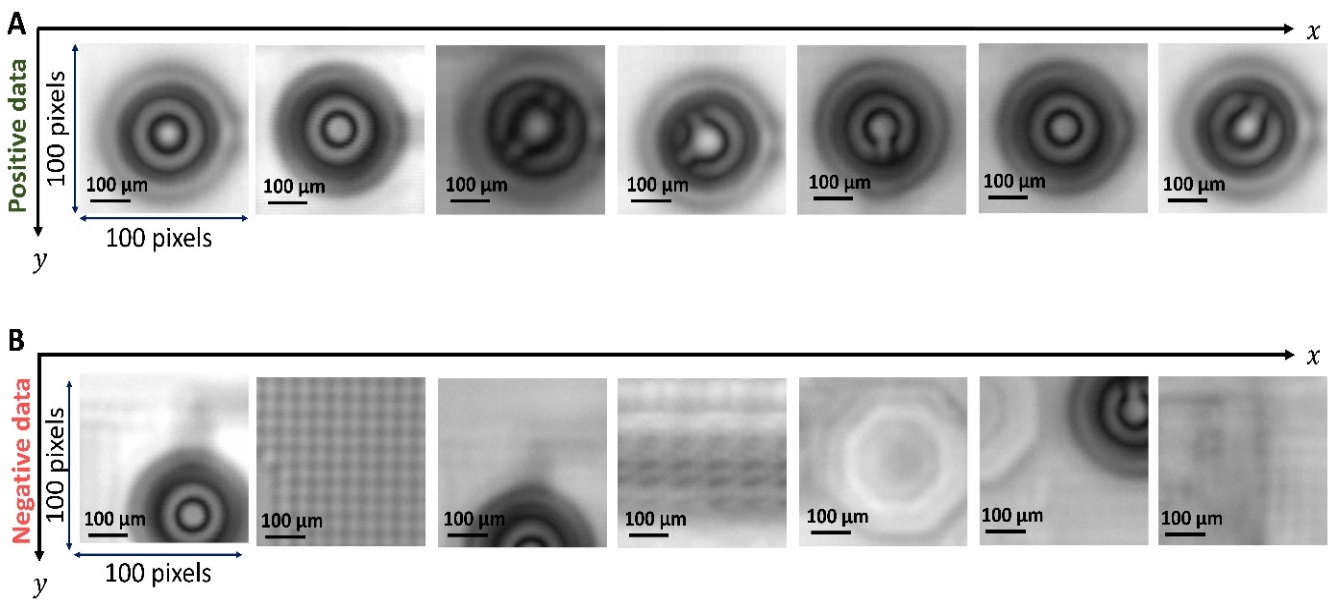
**

**Figure S2. Training data set for TSV localisation. (A)** Positive data with TSVs centred in the image patch. **(B)** Negative data with background and/or TSVs that are not centred.

**
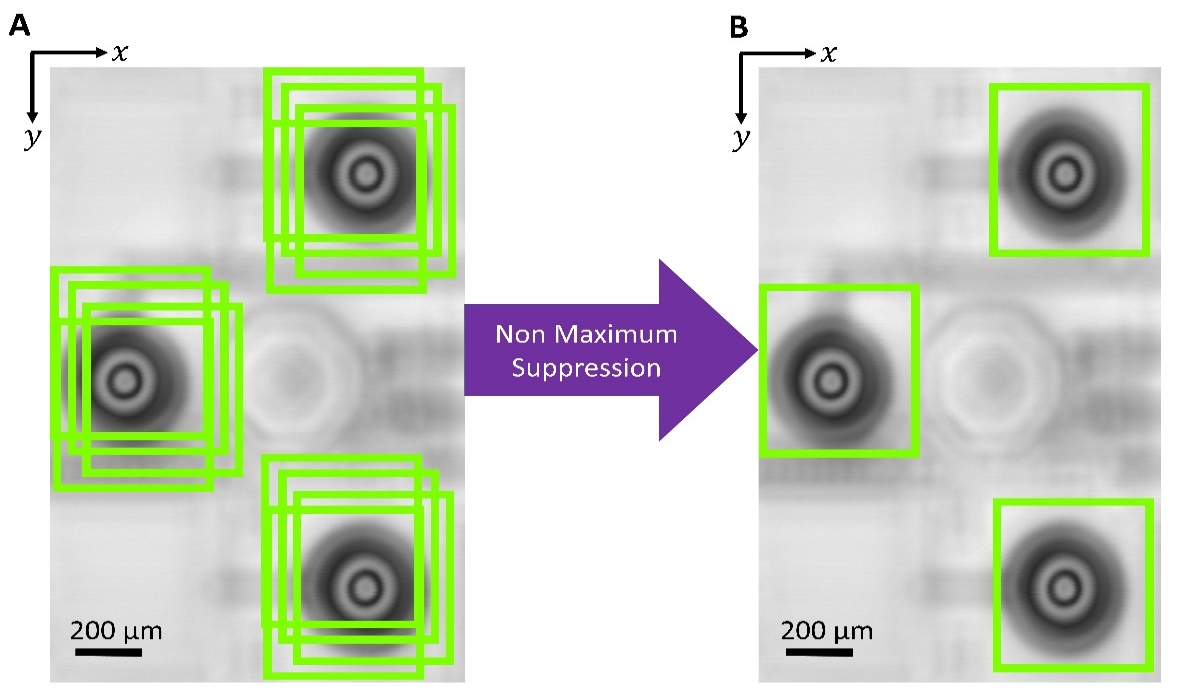
**

**Figure S3. Finding the best bounding box around the TSV using Non Maximum Suppression (NMS). (A)** before applying NMS. **(B)** After applying NMS.

**
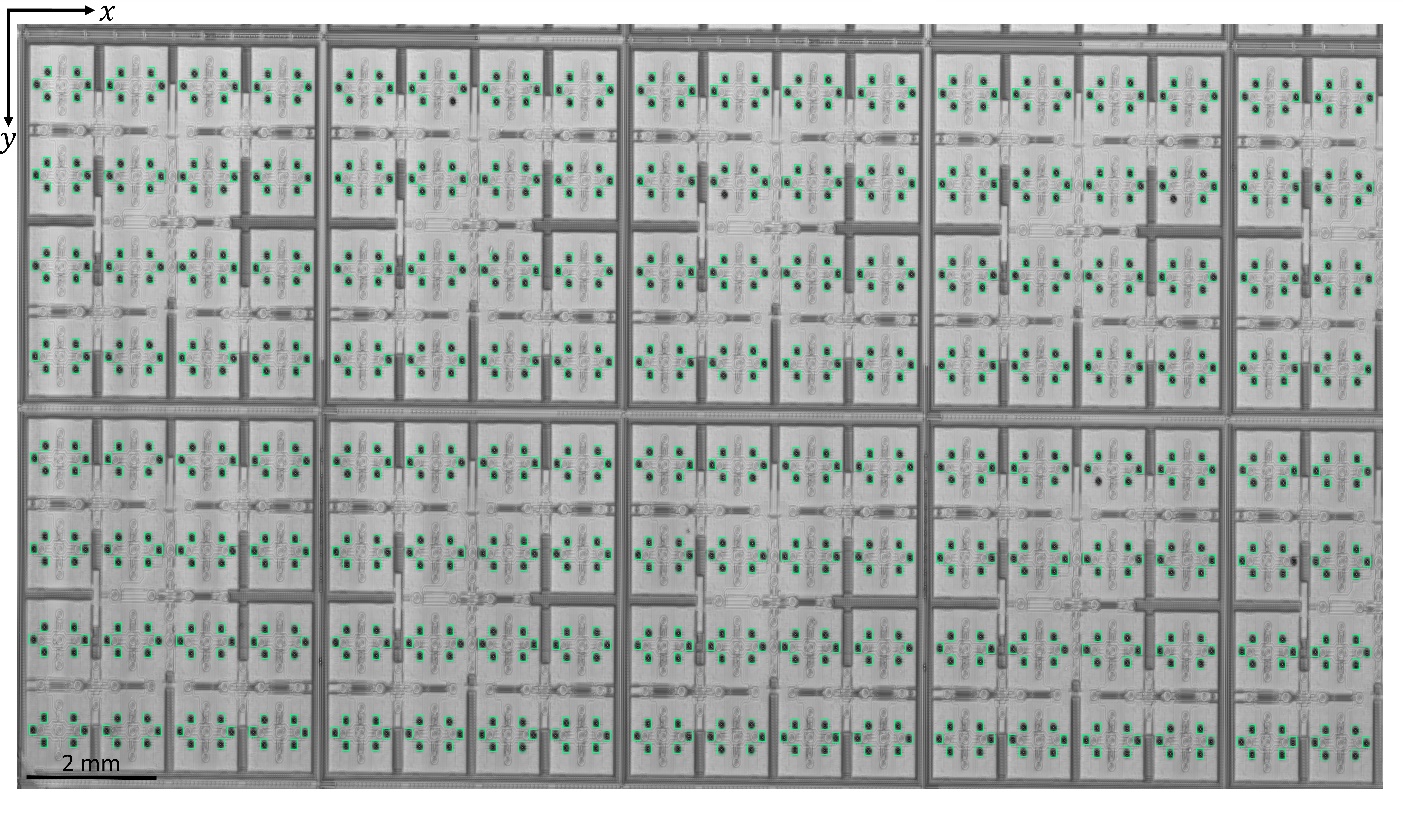
**

**Figure S4. Predictions of sliding window TSV detector using CNN1.** The predictions of CNN1 are shown in ‘chartreuse’ rectangular bounding boxes. Here, we localised 864 TSVs with the characteristic patterns obtained using SAM.


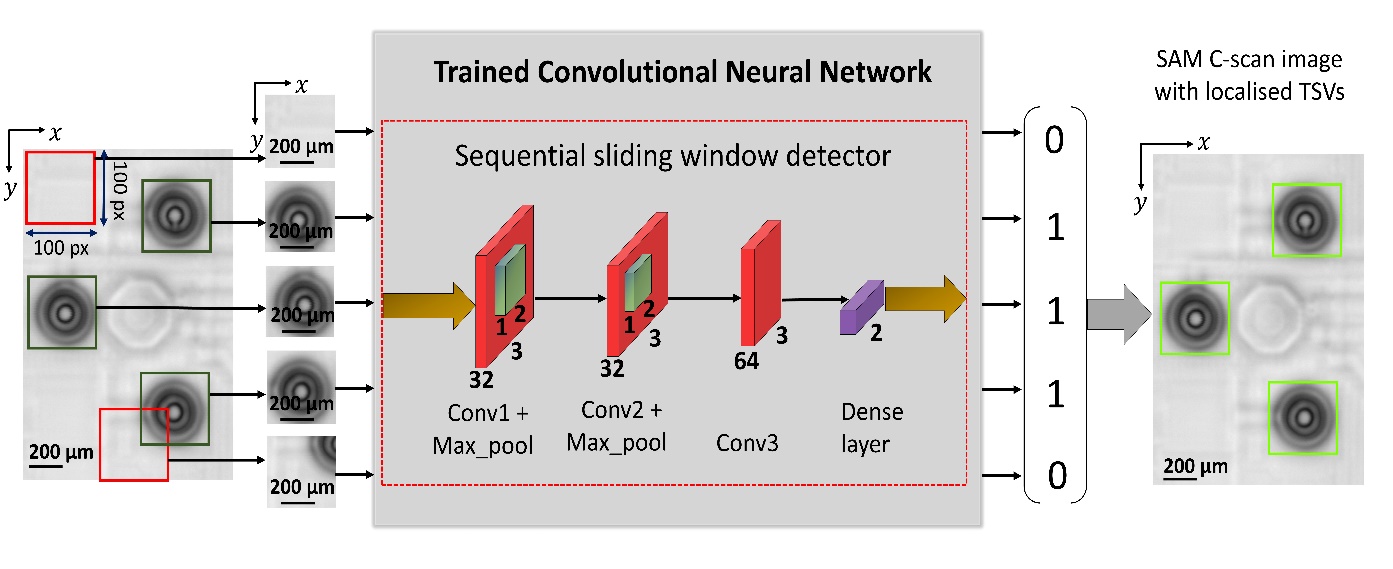
**Figure S5. The Architecture of CNN1 for TSV localisation using sequential sliding window detector.**


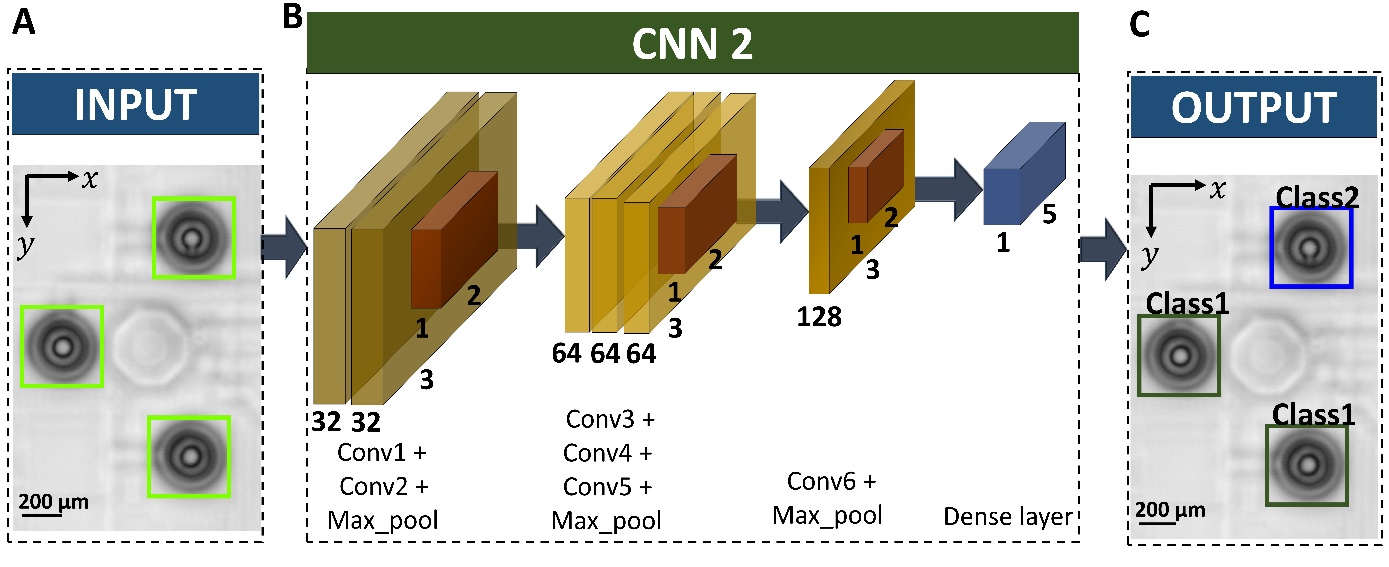


**Figure S6. Architecture of CNN2 for TSV classification. (A)** The predictions by CNN1 are the input to CNN2. These predictions by CNN1 is shown in ‘chartreuse’ rectangular boxes. **(B)** Structure of CNN2. **(C)** The predictions of CNN2 is shown in dark green and blue color rectangular boxes with title 'Class1' and 'Class2' respectively.


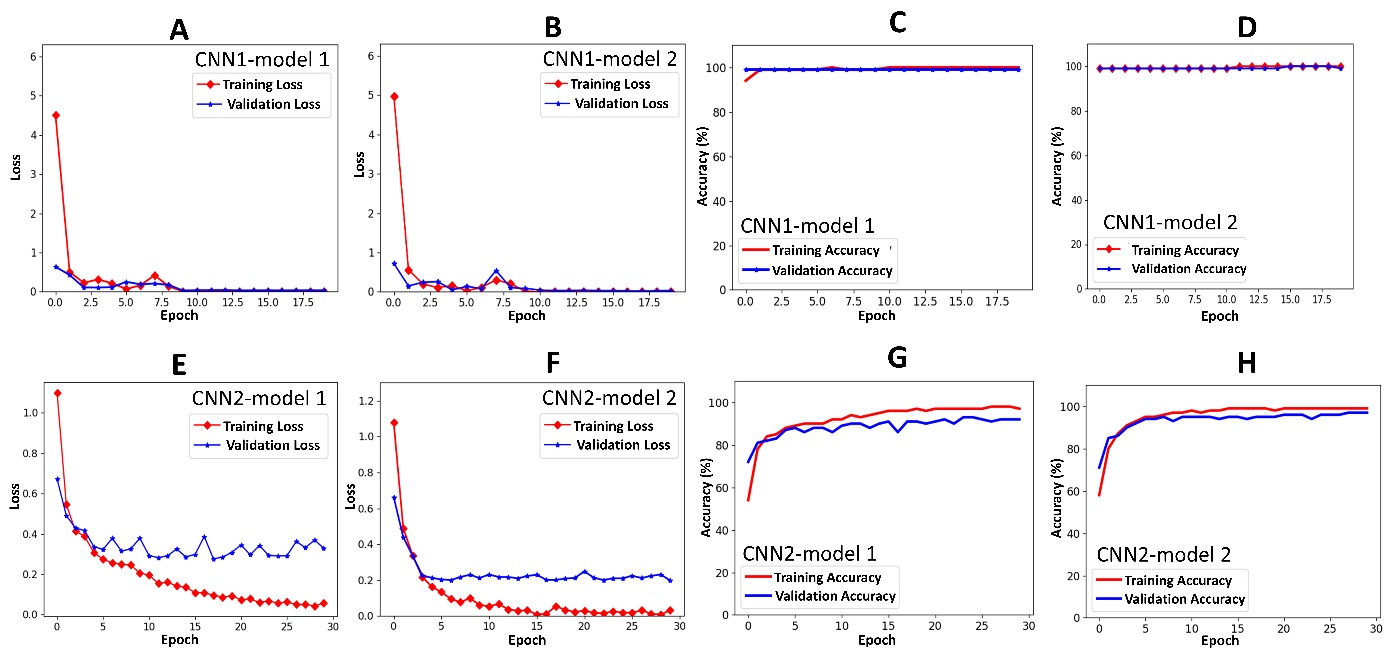


**Figure S7. Comparison of training & validation loss and accuracy curves of different architectures of CNN1 and CNN2.** Training and validation loss curve of CNN 1 using **(A**) model 1 and **(B)** model 2. CNN1 is trained using ‘model 1’ with 32,64 filters and ‘model 2’ with 32,32,64 filters. The accuracy versus epoch plot of CNN 1 using **(C**) model 1 and **(D)** model 2. Model 2 is used for CNN1 throughout the paper due to slightly better validation accuracy. Similarly different architectures for CNN 2 are trained for the TSV classification named model 1(32,32,64 filters) and model 2(32,32,64,64,64,128 filters). Training and validation loss curve of CNN2 using **(E**) model 1 and **(F)** model 2. The accuracy versus epoch plot of CNN2 using **(G**) model 1 and **(H)** model 2. Model 2 is used due to its validation accuracy for CNN2 throughout the paper.


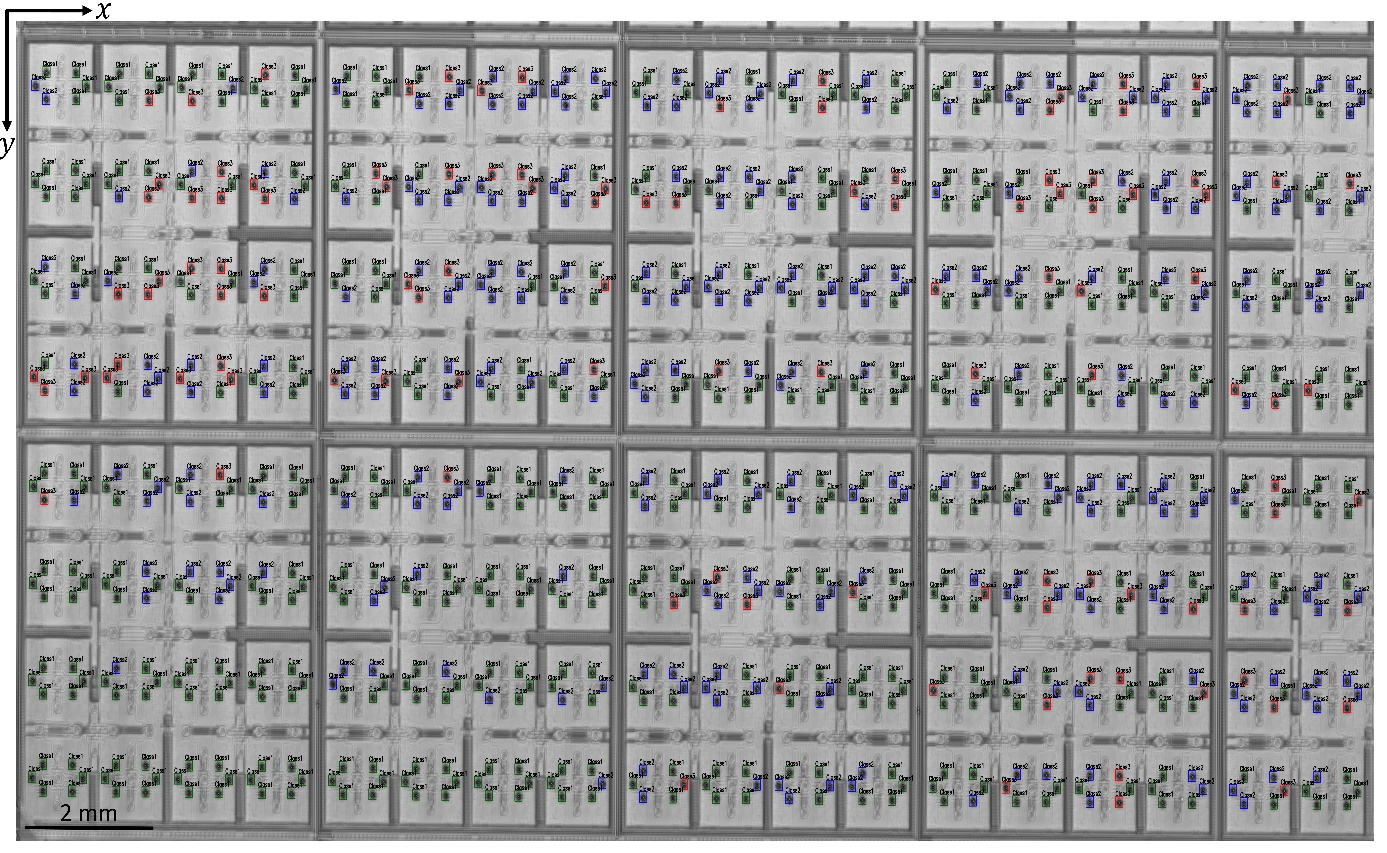


**Figure S8. The prediction result according to E2E-CNN.** The quality predictions of 864 TSVs are represented in dark green, blue and red rectangular boxes according to class 1, 2 and 3.


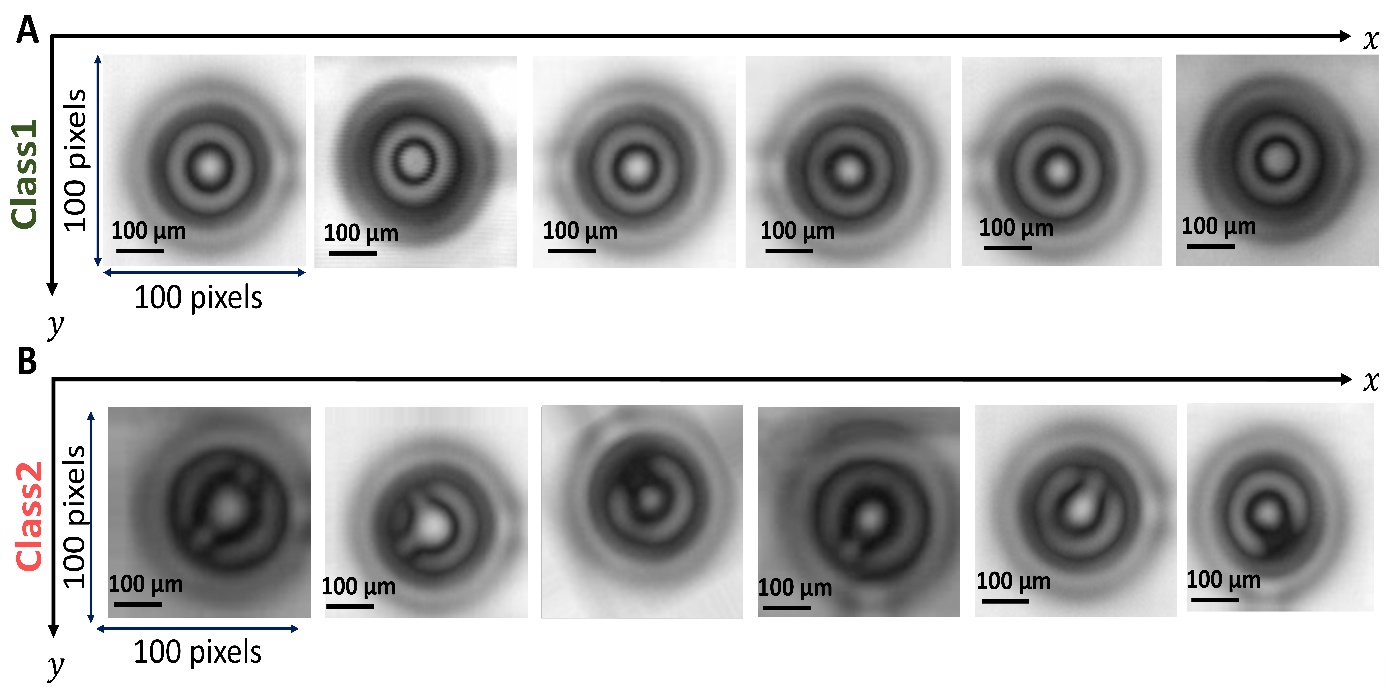


**Figure S9. Data set for semi-automated TSV classification. (A)** SAM C-scan of TSVs with non-disturbed (homogeneous) fringes in the characteristic pattern – Class1. **(B)** SAM C-scan image with disturbed (inhomogeneous) fringes – Class2.


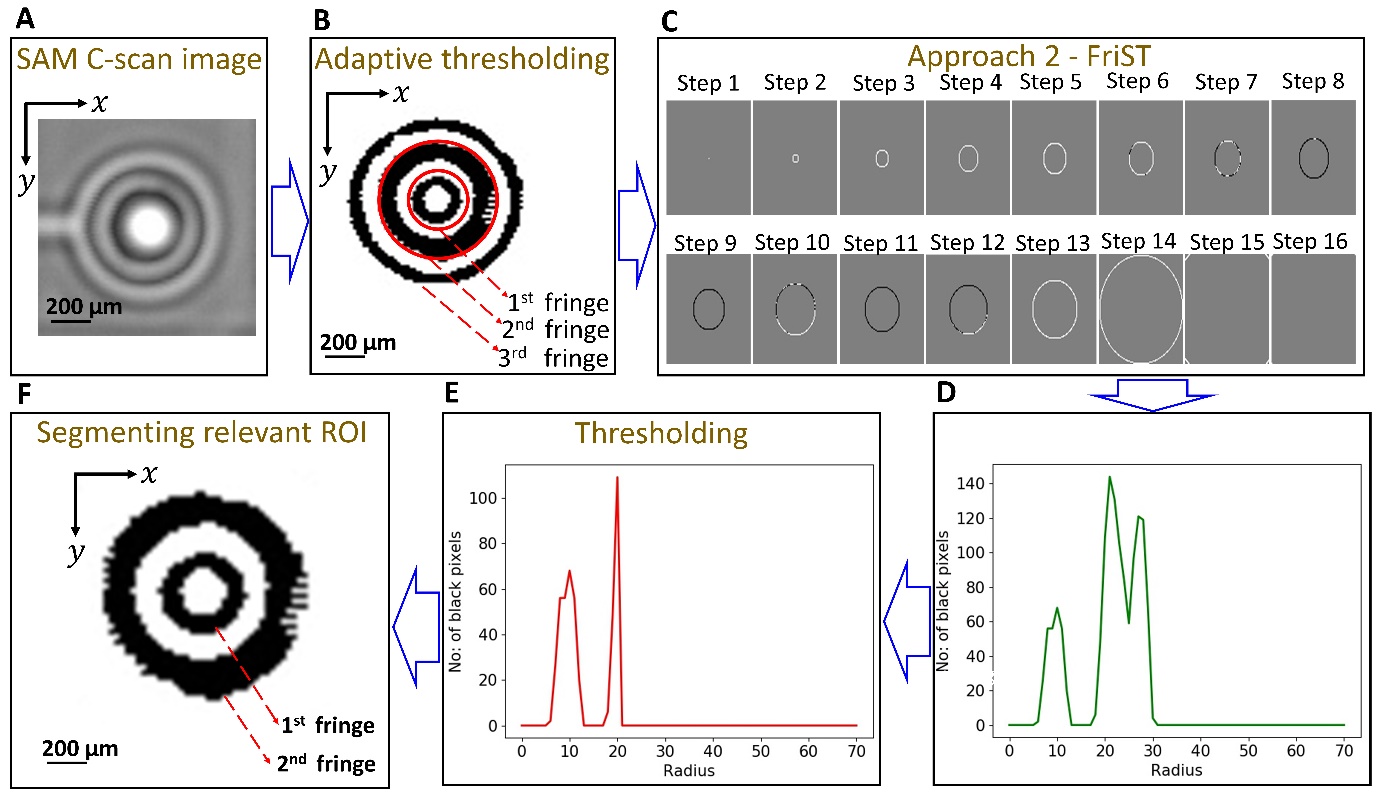


**Figure S10. Feature extraction using Fringe Segmentation Technique (FriST) of a TSV with non-disturbed (homogeneous) fringes. (A)** SAM C-scan image of a single TSV. **(B)** C-scan image of single TSV after adaptive thresholding. **(C)** Images are generated at various steps while increasing the radius of the considered unit circle from the centre of the pre-processed image. **(D)** Plot between the number of black pixels at the circumference of the grown circle versus the radius. **(E)** Thresholding to remove the third fringe from the characteristic pattern. **(F)** The output of FriST segmentation technique.


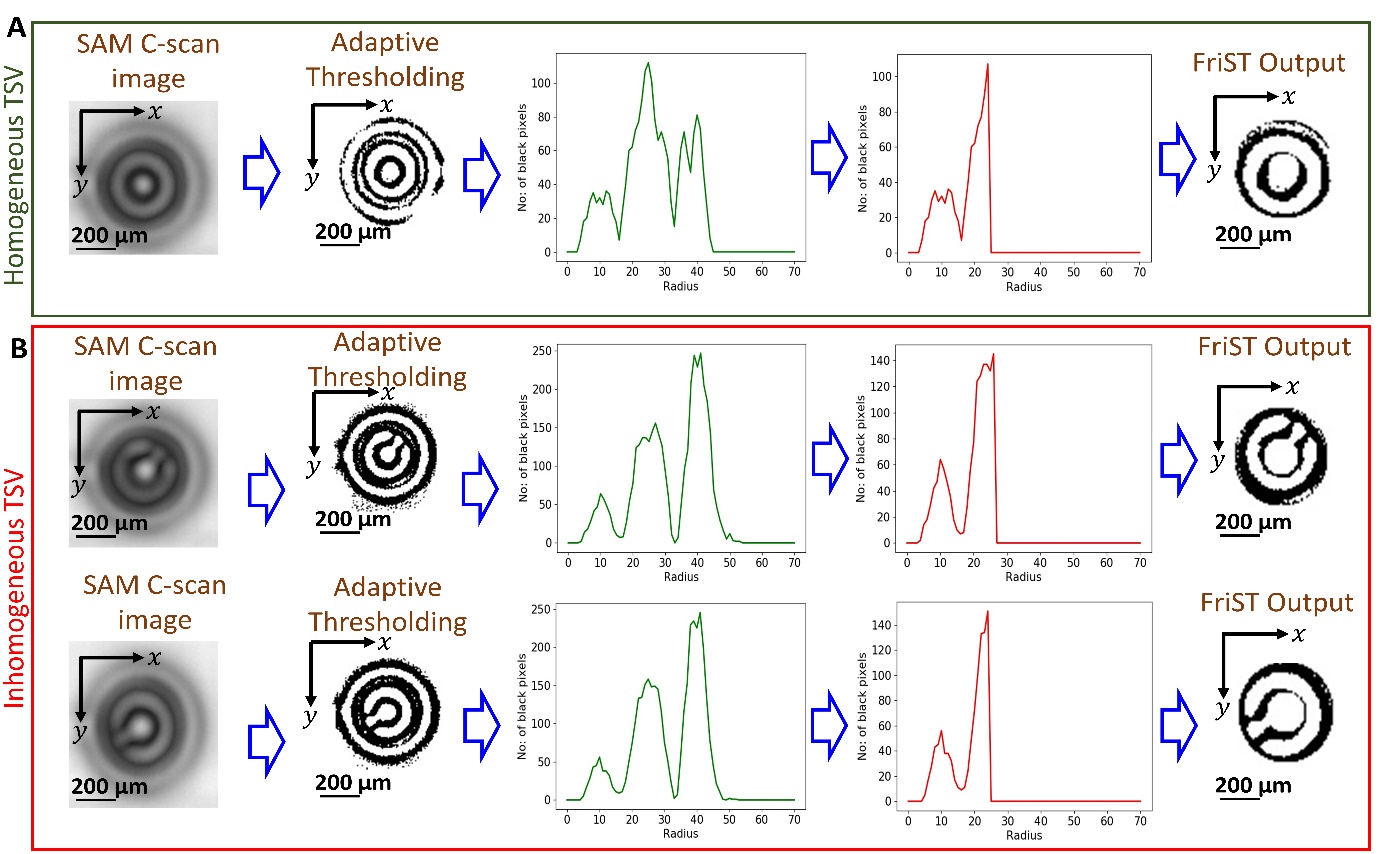


**Figure S11. Feature extraction using Fringe Segmentation Technique (FriST) of TSVs. (A)** with homogeneous fringes. **(B)** Inhomogeneous fringes.


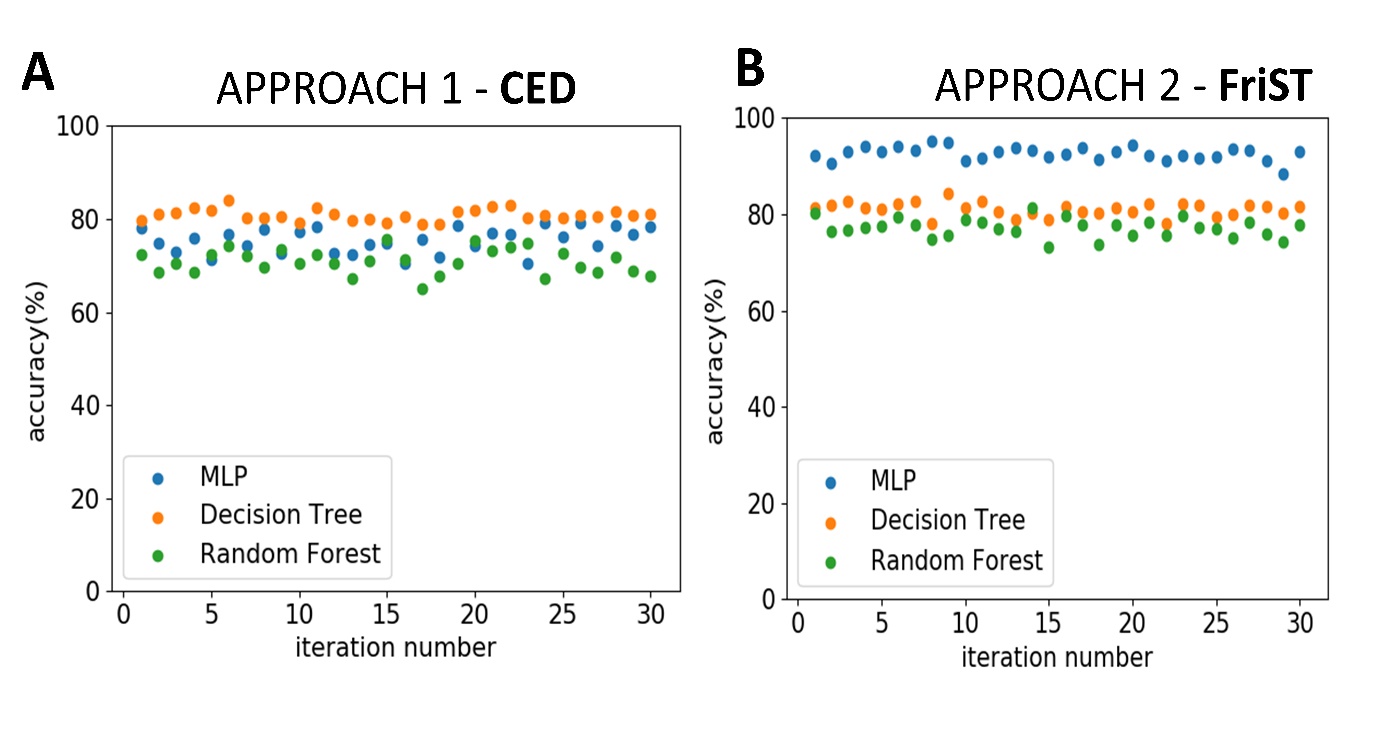


**Figure S12. Semi-automated classification accuracy plots of MLP, DT and RF models using (A)** CED and **(B)** FriST feature extraction techniques.


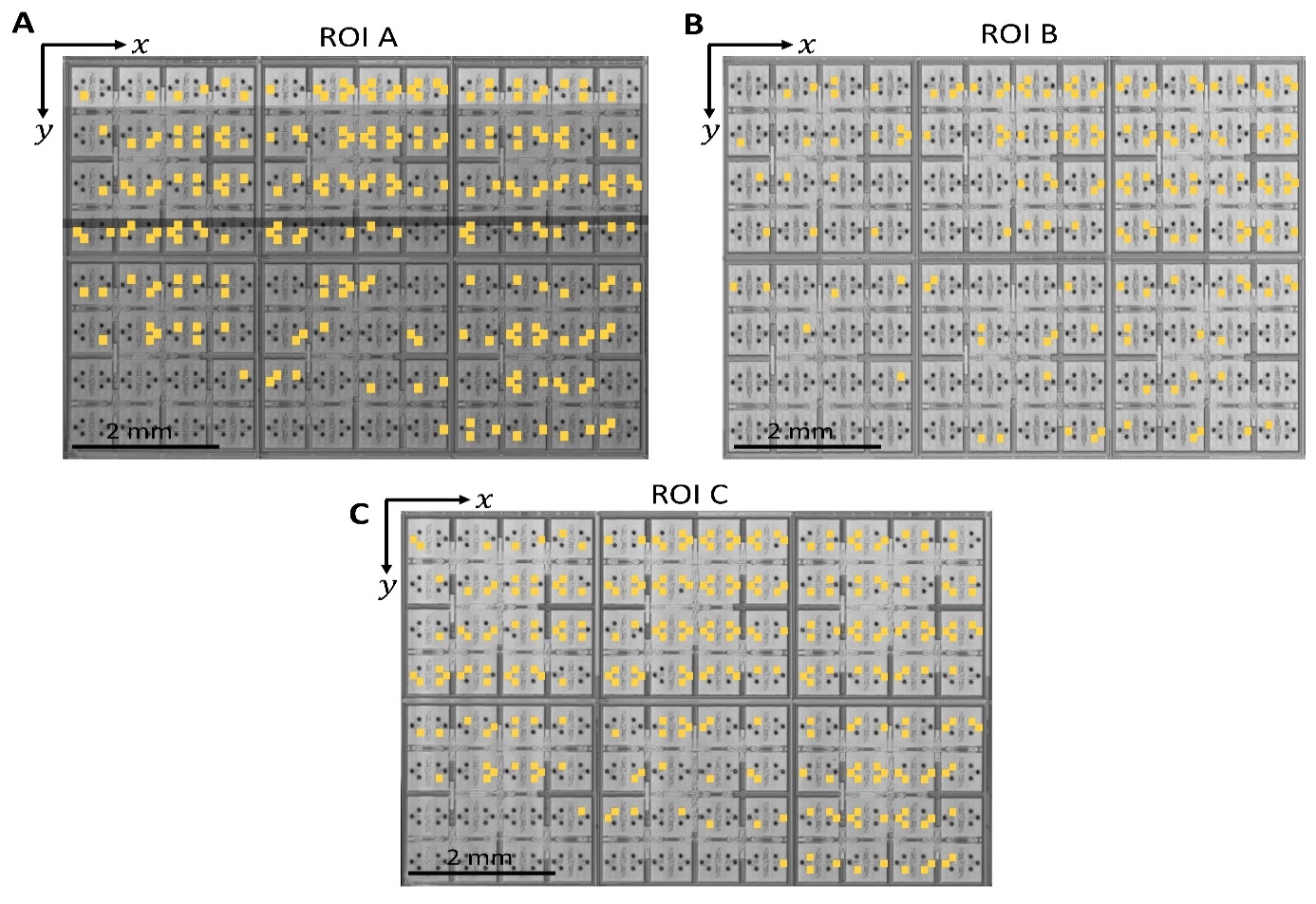


**Figure S13. Localisation and classification of TSVs from SAM C-scan image using E2E CNN. The TSVs that are predicted as defective (characteristic patterns of TSVs with an inhomogeneity in the fringes) by the model are marked in yellow rectangular boxes. (A)** ROI A. **(B)** ROI B. **(C)** ROI C.
